# Enhancing monellin production by *Pichia pastoris* at low cell induction concentration via effectively regulating methanol metabolism patterns and energy utilization efficiency

**DOI:** 10.1371/journal.pone.0184602

**Published:** 2017-10-05

**Authors:** Luqiang Jia, Tingyong Tu, Qiangqiang Huai, Jiaowen Sun, Shanshan Chen, Xin Li, Zhongping Shi, Jian Ding

**Affiliations:** 1 The Key Laboratory of Industrial Biotechnology, Ministry of Education, School of Biotechnology, Jiangnan University, Wuxi, Jiangsu, China; 2 School of Biology and Pharmaceutical engineering, Wuhan Polytechnic University, Wuhan, Hubei, China; Universite Paris-Sud, FRANCE

## Abstract

In heterologous protein productions by *P*. *pastoris*, methanol induction is generally initiated when cell concentration reaches very high density. The alternative strategy by initiating methanol induction at lower cells concentration was also reported to be effective in easing DO control, reducing toxic by-metabolites accumulation and increasing targeted proteins titers. However, the methanol/energy regulation mechanisms are seldom reported. We theoretically analyzed the methanol/energy metabolisms in protein expression process with the strategies of initiating induction at higher or lower cells concentrations, using monellin production as a prototype. When initiating induction at lower cells concentration and controlling induction temperature at 30°C, monellin concentration reached the highest levels of 2.62~2.71 g/L, which was 2.5~4.9 fold of those obtained with the strategy of initiating induction at higher cells concentration. With the desired induction strategy, 1) carbon metabolism ratio directing into the precursors synthesis route for monellin production reached the highest level of 65%, carbon metabolism ratios towards to precursors synthesis and ATP regeneration routes were regulated at relatively balanced levels; 2) monellin synthesis was completely cell growth associated, with the largest associated coefficient and higher specific growth rate; 3) theoretical NADH (energy) utilization efficiency *η* was the highest, and *η* stayed high levels (≥0.8) during most period (89%) within induction phase to supply sufficient energy in supporting monellin synthesis.

## Introduction

Monellin is a protein extracted from the berries of the West African forest plant *Dioscoreophyllum cumminsii* and its relative sweetness is 3,000 times higher than sucrose on weight basis [1]. Monellin has been perceived as a sweet by humans in old world era. Like the other sweet proteins (pentadin, thaumatin, etc.), monellin could also be recognized as a non-carbohydrate sweeter (sweetener) and is particularly beneficial to the diabetics individuals who rely on sugar-intake but suffer from dangerously elevated glucose (blood sugar) levels [2]. This product could be developed into a consolation sweetener or sweet yeast tablets for patients with diabetes [3, 4]. Pure monellin is expensive, Sigma Co. sells monellin with 95% purity at $100/100 mg (http://www.zhenghe.cn/JiShuTong/CG_TechnologyInfoT.aspx?key=24ccc46229984a019028ee1a95034ac3). Monellin could be biochemically produced by microorganisms. In recent reports, Chen et al. showed that up to 0.29 g/L of monellin could be expressed by *Bacillus subtilis* with *sacB* promoter and signal peptide [5]. Liu et al. expressed monellin in recombinant *Saccharomyces cerevisiae* and monellin concentration reached 0.675 g/L [6]. Leone et al. produced monellin by a recombinant *Escherichia coli* and monellin concentration reached about 0.18 g/L [7].

Methylotrophic *P*. *pastoris* is one of the most effective systems for expression of heterologous proteins [8–10], which features with simplicity of easy molecular genetic manipulation; availability to reach high cells density; high proteins expression/secretion abilities, etc. Heterologous proteins production by recombinant *P*. *pastoris* is basically divided into two phases: a growth phase to accumulate a large amount of functional cells with glycerol as the carbon source, and an induction phase by feeding methanol to produce heterologous proteins [11–13]. In heterologous proteins production by *P*. *pastoris*, methanol induction is generally initiated when cell concentration reaches very high density (100~130 g-DCW/L), and then shifting glycerol feeding into methanol feeding and maintaining methanol concentration at an adequate level [14, 15]. However, this production strategy suffers with the following problems: 1) the high oxygen consumption characteristics, pure oxygen has to be aerated to obtain high cell density, which deteriorates the entire fermentation economics; 2) ethanol accumulation during the late growth phase if high cell density is pursued, resulting in the unstable heterologous protein productions [16]; 3) induction at lower temperature (20°C) was reported to be beneficial for heterologous protein productions by enhancing AOX activity and relieving targeted proteins hydrolysis, but it is at the expense of increasing heat exchange and oxygen supply loads/costs particularly in summer season [17]; 4) the inefficient energy (NADH) utilization may cause excessive/inefficient methanol consumption which also deteriorates the entire fermentation performance [11]. To overcome the above mentioned problems, alternative operation strategy by initiating methanol induction at relatively lower cells concentration was proposed. Wang et al. used this strategy for alkaline polygalacturonate lyase production, and they found that, the highest protein productivity (Qv) was obtained when initiating methanol induction at lower cells concentration of 56.7 g-DCW/L. Qv was 11.6% and 18.4% higher than those when initiating methanol induction at 83.39 or 124.9 g-DCW/L, respectively [18]. Jia et al. reported that the enhanced poly (vinyl alcohol) dehydrogenase production was achieved when initiating methanol induction at 60 g-DCW/L while maintaining induction temperature at lower level of 22°C, as the dissolved oxygen concentration (DO) could still be under control (5~20%) [19]. However, reports on methanol/energy metabolism patterns variations when initiating induction at lower/higher cells concentrations are seldom available.

In this study, we analyzed the methanol/energy metabolism patterns in monellin production by *Pichia pastoris* at different cell induction concentrations (50/100 g-DCW/L) and temperatures (20/30°C), attempting to interpret the reasons responsible for enhanced monellin expression achieved by the operation strategy of initiating induction at lower cells concentration. The analysis results would also supply valuable information on process control of other foreign protein productions by *Pichia pastoris*.

## Materials and methods

### Microorganisms

*P*. *pastoris* KM71, a Mut^s^ strain, was used as a host strain for gene expression by ligation monellin gene into pPICZaA [20]. The recombinant *P*. *pastoris* was provided by School of Biology and Pharmaceutical engineering, Wuhan Polytechnic University, China. In addition, recombinant *P*. *pastoris* GS115, a Mut^+^ *P*. *pastoris* strain expressing human serum albumin-fibroblast growth factor 21 fusion protein (HSA-FGF21) was also used in supporting the advantages/universal ability of the desired operation mode. This strain was kindly provided by Pharmaceutical School, Wenzhou University, China.

### Media

The composition of the media (in g/L) was as follows: YPD medium (glucose 20, yeast extract 10, peptone 20) was used for seed culture. The medium for batch fermentation: glycerol 20, (NH_4_)_2_SO_4_ 5, H_3_PO_4_ 2 (%, v/v), MgSO_4_ 1, CaSO_4_ 0.1, K_2_SO_4_ 1; PTM1 [21] 10 (mL/L), pH 6.0. Feeding medium for cells growth: glycerol 500, (NH_4_)_2_SO_4_ 0.5, KH_2_PO_4_ 0.5, MgSO_4_ 0.03; PTM1 10 (mL/L), pH 6.0. Feeding medium for induction: pure methanol, PTM1 10 (mL/L), pH 6.0.

### Monellin expression by *P*. *pastoris* fed-batch cultivations in 5L bioreactors

The fed-batch culture was implemented in a bioreactor (BLBIO-5GJ-3-H, Bailun Bio Co., China) with a monitor/control cabinet unit to run three (3) 5L bioreactors simultaneously. The initial medium was 2.3 L. Inoculation and aeration rate were 14% (v/v) and 3 vvm. In cells growth phase, DO was maintained above 10% by manually/consecutively raising agitation rate until 700 rpm. The standard or modified DO-Stat [16] strategies were used for glycerol feeding, to allow cells concentration to reach a high density level. Pure oxygen was aerated if DO base line could not be maintained above 10% under the maximum agitation rate. pH was maintained at 6.0 by adding 5% (v/v) ammonia water. The induction was initiated by feeding pure methanol after glycerol/ethanol (by-product) was completely consumed and the induction temperature was kept at either 30°C or 20°C upon requirement. pH was controlled at 6.0. An industrial computer equipped with a multi-channels A/D-D/A converter (PCL-812PG, Advantech Co., Taiwan) drove a peristaltic pump (BT00-50M, Langer Co., China) to feed methanol or glycerol based on the on-line measurements of the methanol electrode (FC-2002, Subo Co., China) and DO probe. In the induction phase, methanol concentration was controlled in a range of 5~7 g/L. This methanol electrode could also be used for on-line ethanol measurement during growth phase to adaptively regulate glycerol feeding rate combined with DO measurement [16]. The O_2_ and CO_2_ partial pressures in exhaust gas (air aeration) were on-line monitored by a gas analyzer (LKM2000A, Lokas Co., Korea), and then O_2_ uptake rate (OUR) and CO_2_ evolution rate (CER) were determined using the standard calculation formula [22].

### Measurements of cell/methanol/monellin concentrations and relevant growth/consumption/production rates

The cells concentration was gained by measuring the optical density at 600 nm (OD_600_), then dry cell weight (DCW/L) was calculated by a consistent calibration curve of DCW (DCW/L = 0.25×OD_600_). Methanol and ethanol were measured by a gas chromatography (GC112A, FID detector, Shanghai Precision & Scientific Instrument Co., China) with an Alpha-Col AC20 capillary column (SGE Int’l Pty. Ltd., Australia). The intermediate metabolites of formaldehyde and formate were measured with a HPLC equipped with a reverse-phase ZORBAX SBAqC18 column and detected at 254 nm with an UV detector. The mobile phase contained 99% 20 mmol/L Na_2_PO_4_ solution plus 10% acetonitrile. The injection volume was 10 μL and the column temperature was set at 28°C. For monellin concentration measurement, an amount of 20 μl sample was placed in each lane of the electrophoretic plate. The SDS-page electrophoresis (12% resolving gel) was performed with the molecular weight standards until the bromophenol blue marker had reached the bottom of the gel. After SDS-page analysis, the monellin concentration was quantified by a G: Box Bio Imaging System and GeneTools software (SynGene Co., Cambridge, UK). The intensity of each band (monellin) was scanned in triplicate and the average of three readings was obtained. The cells-free supernatant was used as a crude sample for the sweetness test. A 10 g/L sucrose solution was prepared for comparison. The sweet taste and sensory test of the monellin was performed by the volunteer testers. An electronic balance (JA1102, Haikang Instrument Co., China) connecting with the industrial computer via RS232 communication cable was used for on-line monitoring the methanol/glycerol consumption amounts (g/L), by measuring the weight losses of methanol/glycerol feeding reservoirs. The cells concentrations, methanol/glycerol consumption amounts and monellin concentrations were smoothed by the corresponding quadratic polynomials with (induction) time as the independent variable, and then the cells growth, glycerol consumption, methanol consumption and monellin production rates at certain instant were determined by differentiating the concentrations/amounts with regard to time *t*. The enzymatic activities of AOX (alcohol oxidase), FLD (Formaldehyde dehydrogenase) and FDH (Formate Dehydrogenase) were measured using the method described by the literatures [23, 24].

### RNA purification, cDNA synthesis, and real-time fluorescence quantitative PCR analysis

The transcriptional levels of the four key genes, *aox2*, *fld1*, *fdh1* and *das1* were measured by KangChen Bio-tech, Shanghai, China. Total bacterial RNA was extracted using Trizol Plus RNA Purification Kit (Invitrogen^™^) and purification method described in the manual of the kit. Total RNA was used as the template to synthesize cDNA, and then cDNA products were amplified by the method of real-time fluorescence quantitative PCR. Primers of β-actin template and key enzymes genes were: *β-actin* (F:5'AGGTTCCCACTTATTTCCC3', R:5'CGAGTATCCTCCTCAGTTT CC3'); *aox2* (F:5'GAGCAACTGAATCCCAAGGTA3', R:5'TTGTCGTGGTTTCTCAT CGTA3'); *fld1* (F:5'GCTGAGTTTGTCCGTATCCC3', R:5'TGGCAACTGAGTCTCC CTT3'); *fdh1* (F:5'AAGGTAAGACCATCGCAACA3', R:5'CATTGACGGTAACAAC ATCG3'); *das1* (F:5'GCTCACGGTTCTGCTCTTGG3', R: 5'GCTCGGTTGGTTTGTC CTG3'). The following PCR conditions were adopted: an initial denaturation step at 95°C for 10 min, followed by an amplification and quantification program repeating for 40 cycles (95°C for 10 s, 60°C for 60 s with a single fluorescence measurement), and a melting curve program (a continuous fluorescence measurement raising temperature from 60 to 95°C with a slow heating unit).

## Results and discussion

### Enhancing monellin production by initiating induction at lower cells concentration

**Fig 1:**
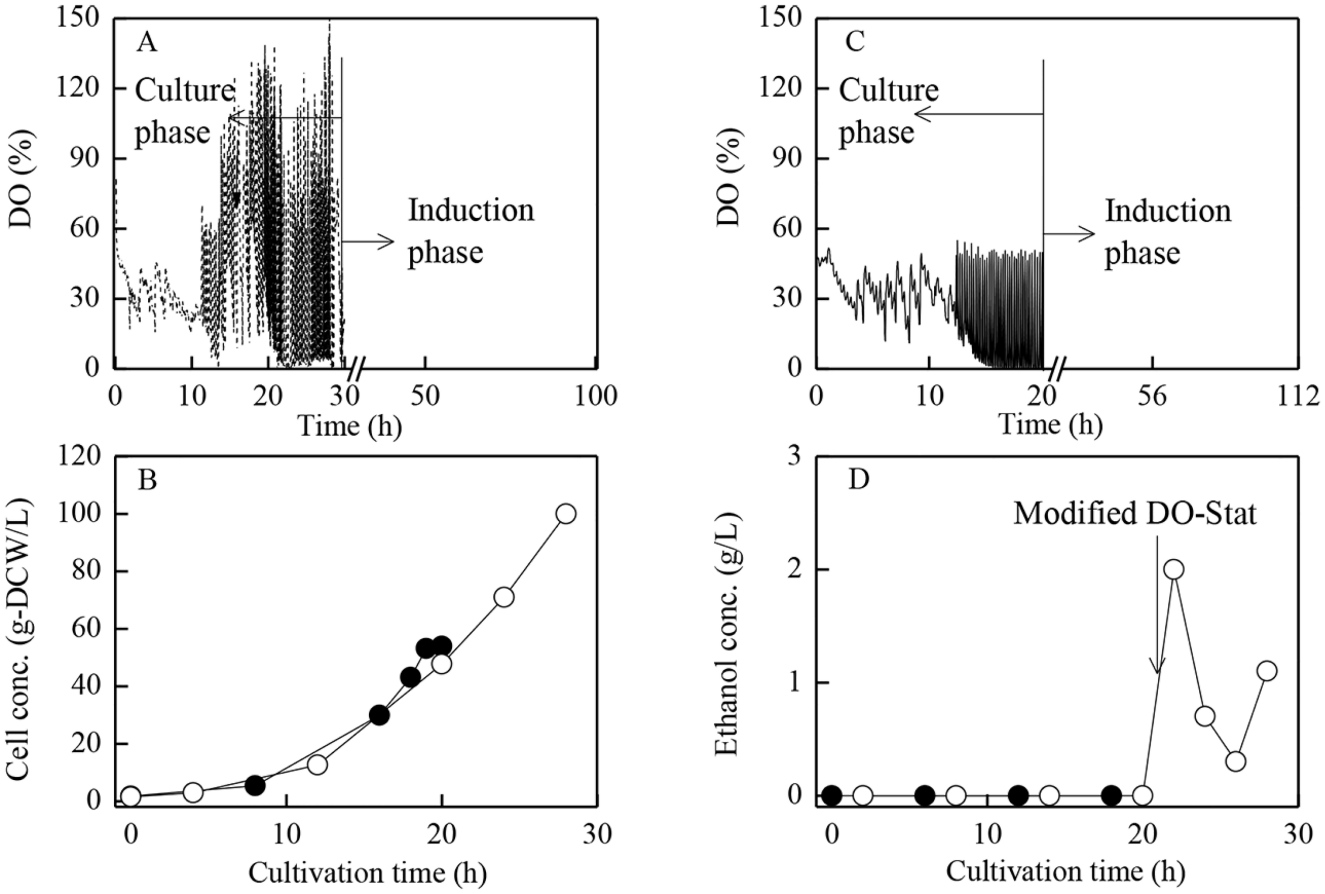
Time courses of DO, cell and ethanol concentrations during cells growth phase under different strategies.

Eight (8) fermentation runs were conducted in the bioreactors. In runs #1–2, methanol induction was started after high cell density (about 100 g-DCW/L) was achieved, while in runs #3 and #6, induction was initiated after cells concentration reaching a relatively lower (about 50 g-DCW/L) level. Fig 1 depicted the fermentation curves (DO, concentrations of cells and ethanol) during the growth phase for run #1 and #3. The standard DO-Stat method was used for glycerol feeding under the condition of air aeration. However, when cells concentration reached more than 50~60 g-DCW/L, DO low-limit (base line) continuously stayed at very low level and the specific cells growth rate declined to near zero level. In run #3, when cells concentration could not further increase, then fermentation shifted from growth phase into induction phase at that particular moment. On the other hand, in run #1, when cells concentration reached about 50 g-DCW/L and cells growth tended to stop, then pure oxygen was directly aerated to replace air. As shown in Fig 1, in this case, cell growth recovered since average DO levels were raised. However, ethanol began to accumulate at that moment. It has been reported that ethanol accumulation would deteriorate heterologous proteins expression by *P*. *pastoris* and the fermentation stability [16]. As a result, a modified DO-Stat strategy [16] was adopted by adaptively adjusting the length of glycerol feeding period, which was basically to regulate average glycerol feeding rate via re-assimilating the accumulated ethanol as an alternative substrate to maintain the ethanol concentration under certain low level (2 g/L). As shown in Fig 1, with pure oxygen aeration and the modified DO-Stat strategy, cells could reach high density (100 g-DCW/L) while ethanol was repressed at a low level of 2.13 g/L. Therefore, high monellin production in run #1 was thus expected.

**Fig 2:**
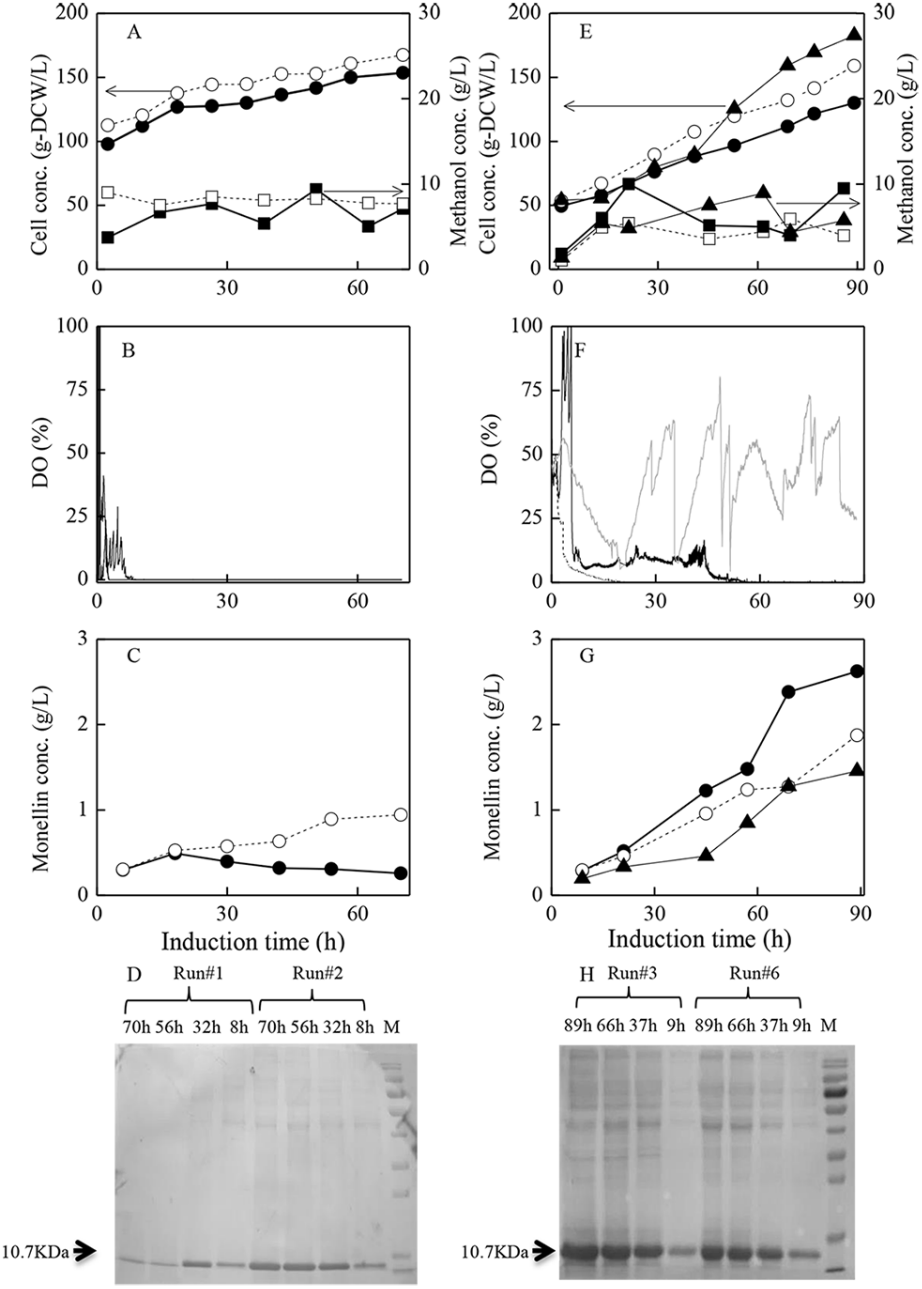
Curves of fermentation data and monellin synthesis/SDS analysis results during induction phase with different strategies.

Fig 2 showed the fermentation results in runs #1–3, #6–7 with different induction strategies during induction phase. In runs #1–2, the standard strategy of initiating methanol induction at higher cell density (~ 100 g-DCW/L) while maintaining induction temperature at 30°C (run #1) and 20°C (run #2) was carried out. On the other hand, in runs #3 and #6, the strategy of initiating methanol induction at lower cell density (~ 50 g-DCW/L) while maintaining the induction temperature at 30°C (run #3) and 20°C (run #6) was implemented. The highest monellin concentration of 2.62 g/L was obtained in run #3, followed by monellin concentration of 1.87 g/L in run #6 (Fig 2G). On the contrast, with the standard induction strategy of initiating methanol induction at higher cells concentration, monellin concentration ended at lower levels of 1.04 g/L and 0.54 g/L (run #2, 20°C) and (run #1, 30°C) respectively (Fig 2C), which were far beyond the expectation. In addition, the cell-free supernatants collected at fermentation end were used to test their sweetness. The double-blind taste test results indicated that the supernatant sweetness obtained in run #3 was much higher than those obtained in other runs. Fig 2D and 2H indicated monellin expression intensity along with the induction time in runs #1–3 and #6, visualized by SDS-PAGE analysis. The band at 10.7 KDa represented monellin. The monellin bands intensity in runs #3 and #6 gradually enhanced along with time, while those in runs #1–2 almost remained unchanged. In the preliminary experiments, we found that the adjusted volumes (bands intensity or densitometry) have close linear relationship with proteins concentrations, using the G: Box Bio Imaging System and GeneTools software. The monellin concentration could thus be determined by monellin adjusted volume/total marker adjusted volumes × protein marker’s concentration, if the same dosages of the marker and fermentation sample were injected. Total protein concentration in protein marker (Tianen Biotech Co. Ltd., Beijing, China) was quantified as 1.835 g/L using coomassie brilliant blue G-250 method. As an example, for the SDS-page lane for run #3 at 89 h, the monellin adjusted volume (bands intensity/densitometry) was 251 (mm^2^) while that of the total adjusted volumes of the marker proteins was 176, therefore monellin concentration was determined as 2.62 g/L. We believed that the analysis method was both reliable and accurate. On the other hand, the intermediate metabolites of formaldehyde and formate were not detected, which indicated non-accumulation of the toxic intermediate metabolites during all fermentation runs (data not shown).

Furthermore, we did the following two runs (run #4–5). Run #4 was a duplicated/repeated experiment of run #3, which was used to show the reproducibility of run #3 (optimal/desired operation mode). The final monellin concentration in run #4 reached a high level of 2.71 g/L, which was at the competitive level of that in run #3 (2.62 g/L). Run #5 was a chemostat fermentation which was carried out by initiating induction at about 50 g-DCW/L and 30°C, then controlling cells concentration around 55 g-DCW/L to maintain the specific cells growth rate (*μ*) at higher level (0.016 h^-1^). Run #5 was attempted to achieve stable/long term’s monellin production and higher productivity simultaneously. All of these results were summarized in Table 1.

**Table 1:** The fermentation performance comparisons of monellin production by *Pichia pastoris* with different induction strategies.

Run#	Induction Temp. (°C)	Initial DCW* (g/L)	Induction time (h)	Final DCW (g/L)	Max monellin conc. (g/L)	Ave. MeOH conc.(g/L)	Ave. DO (%)	Aeration Mode
1	30	97.5	70	153.0	0.54	∼5.0	∼0	Air
2	20	106.0	70	167.0	1.04	∼5.0	∼0	Air
3	30	55.6	89	130.0	2.62	∼5.0	0∼10	Air
4	30	51.5	89	132.0	2.71	∼5.0	0∼10	Air
5^†^	30	55.8	89	92.5	2.16^†^	∼5.0	0∼10	Air
6	20	53.4	89	159.0	1.87	∼5.0	∼0	Air
7	30	53.5	89	114.0	1.65	∼5.0	10∼60	Oxygen
8	20	54.0	89	182.0	1.46	∼5.0	10∼60	Oxygen

1) DCW: Dry Cells Weight; MeOH: Methanol.

2) *: Cell concentration when methanol induction was initiated.

3) ^†^: run #5, the chemostat fermentation, monellin concentration was estimated by Eq 7.

### The basic infrastructure of methanol metabolism in monellin production by *P*. *pastoris*

**Fig 3:**
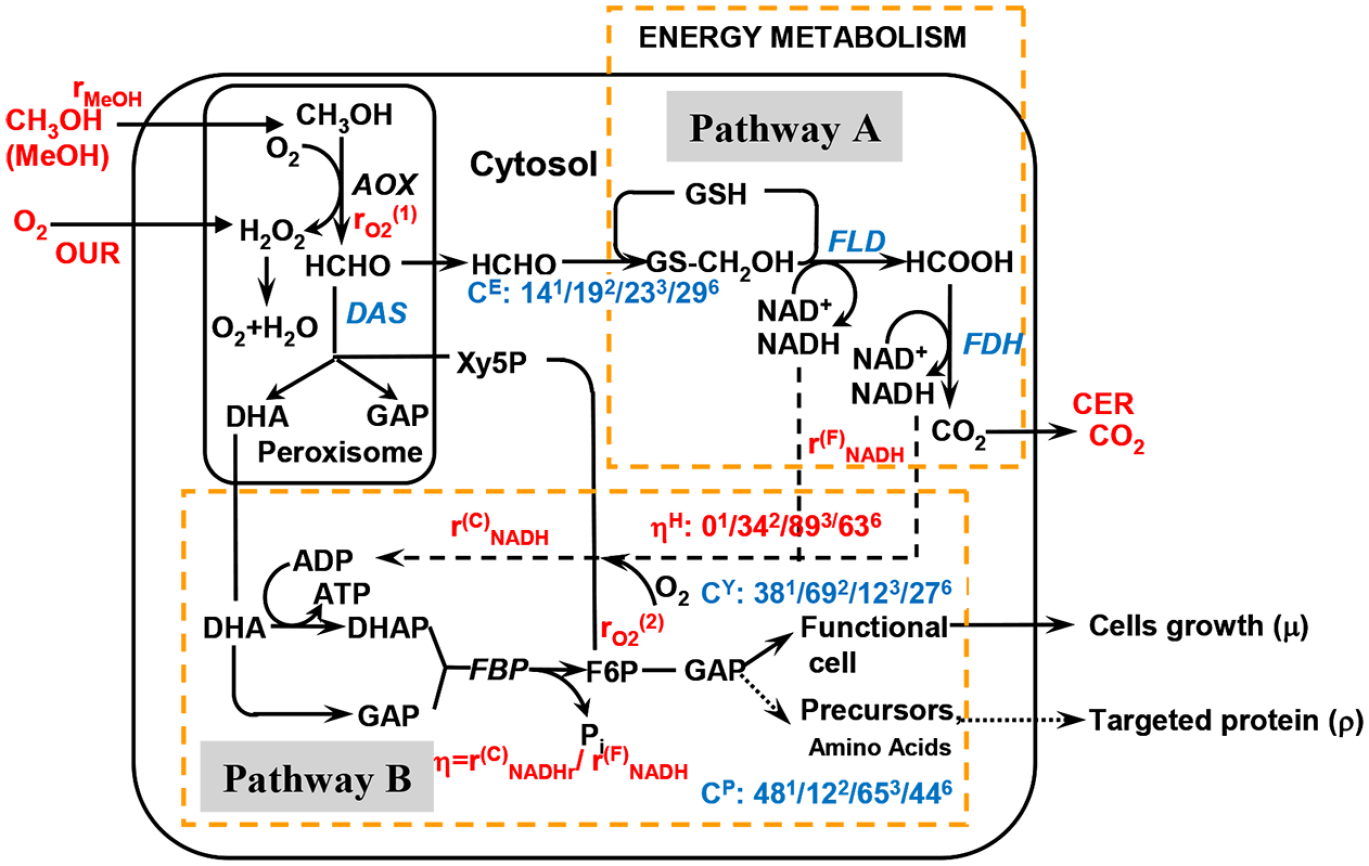
A simplified carbon/metabolic map and carbon/energy distributions for monellin synthesis by *P*. *pastoris*.

Fig 3 indicated the simplified methanol metabolism pathway based on the literature [8], which basically consists of two metabolic pathways: a portion of formaldehyde (HCHO) generated by AOX leaves peroxisome and is further oxidized to formate (HCOOH) and eventually CO_2_ (pathway A). The role of pathway A is to supply a source of energy (NADH) for metabolism. The remaining formaldehyde is assimilated to grow the recombinant cells and to produce the precursors (amino acids, etc.) for synthesis of the targeted foreign proteins by a cyclic pathway (pathway B) via a series of reactions by consuming ATP regenerated [25, 26]. Basically, the entire methanol metabolism contains two portions, one for carbon metabolism and the other for energy formation. The two portions should co-operatively work together in order to efficiently run the entire foreign protein expression system. Çelik et al. [27] also showed that when using methanol as the sole carbon/induction source, methanol metabolism at G3P note would be enhanced and the metabolic pathways of gluconeogenesis, glycolysis and pentose phosphate be activated, which promote the precursors production (nucleotides and amino acids including Pro, Arg, Asp, Ala, Val, Leu, Ile, Phe, Tyr, etc.) for foreign protein synthesis and cells constituents.

### Carbon metabolism patterns in monellin production by *P*. *pastoris* under different induction strategies

As shown in Fig 3, carbon (methanol) metabolism could be divided into four (4) different portions: cells growth, maintenance, monellin synthesis and energy formation. Carbon distribution ratios for cells growth and metabolism maintenance could be determined by the following well-recognized equation.

$$v=\frac{1}{Y_{X/S}}\mu +m$$ (1)

where *ν*, *μ*, *Y*_X/S_ and *m* represented specific methanol consumption rate, specific cells growth rate, cells yield on methanol and maintenance coefficient. By depicting *ν* versus *μ*, methanol distribution ratios for cells growth and maintenance under different induction strategies could be determined. On the other hand, CO_2_ has to be released in pass A by consuming a portion of methanol, to produce energy substance (NADH) in support of the assimilation route (pass B) as shown in Fig 3. This energy consumption ratio *ε* could be calculated by Eq 2.

$$\varepsilon =\frac{\int_{0}^{t_{f}}CER(t)dt}{A_{MeOH}^{T}}$$ (2)

where CER, *t*_f_ and *A*^T^_MeOH_ represented CO_2_ evolution rate, total induction time and methanol consumption amount. Then, the methanol utilization ratio for monellin synthesis (*γ*) could be determined by:

$$\begin{array}{l} \gamma =1-Y_{X/S}-\varepsilon \\ \varepsilon =\varepsilon_{P}+m_{XE} \end{array}$$ (3)

Here, we separated the carbon metabolic ratios for supplying energy (*ε*) in two parts: *ε*_P_ and *m*_XE_. *ε*_P_ and *m*_XE_ represented carbon distribution ratio for the energy (NADH) required in monellin synthesis/cells growth route and pure cells maintenance, respectively. Then, we re-formulated Eq 1 into Eq 4 to obtain the dimensionless variable *m*_XE_:

$$\begin{array}{l} \frac{\mu }{\nu }=Y_{X/S}-\frac{mY_{X/S}}{v}=Y_{X/S}-\frac{k}{v}=Y_{X/S}-m_{XE}\\ m_{XE}=\frac{k}{\nu } \end{array}$$ (4)

*m*_XE_ was a dimensionless variable because *m* and *ν* have the same dimensional unit (h^-1^). However, *m*_XE_ was not a constant value any longer but a variable as 1/*ν* and *μ*/*ν* varied, we used the average value in determination of *m*_XE_:

$$m_{XE}=\bar{m_{XE}}=\frac{\Sigma \left(m_{XE}(t)\Delta T\right)}{T}$$ (5)

where *m*_XE_(t), Δ*T* and *T* referred to *m*_XE_ at particular induction instant, sampling interval and total induction time. Furthermore, *Y*_X/S_ determined by Eq 1 was an “apparent” yield. Literatures indicated that carbon element weight content occupies about 50% in *P*. *pastoris* cells [18, 28]. Thus, we used “apparent” *Y*_X/S_ × 0.5 to determine the real *Y*_X/S_ (Table 2) and *ε*_P_ (Eq 3, Table 2).

**Table 2:** Major secondary fermentation parameters in monellin production by *Pichia pastoris* with different induction strategies.

	Carbon Distribution Ratios (%)				Monellin Synthesis Parameters (×10^−5^)		*η* Distribution (%)	
Run#	Cells Growth *Y*_X/S_	Maintenance *m*_*XE*_	Energy *ε*_*P*_	Precursors *γ*	*α*	*β*	*η*>0.8	0≤*η*≤0.8
1	18.9	19.3	14.0	47.8	-	-	0.0	100.0
2	29.3	40.2	19.0	11.5	610	4	33.9	66.1
3	12.1	0.0	22.7	65.2	3,650	0	88.8	11.2
4	15.1	5.8	27.2	51.9	3,080	0	78.6	21.4
5	-	-	12.8	-	-	-	74.6	25.4
6	16.5	10.1	29.2	44.2	1,590	0	62.8	37.2
7	-	-	-	-	2,340	3	-	-
8	-	-	-	-	1,370	0	-	-

1) runs # coincided with those in Table 1

2) “-” meant not available.

In addition, we assumed that specific monellin synthesis rate (*ρ*) and cells growth rate (*μ*) follows Luedeking-Piret model, that is,

ρ=αμ+β (6)

**Fig 4:**
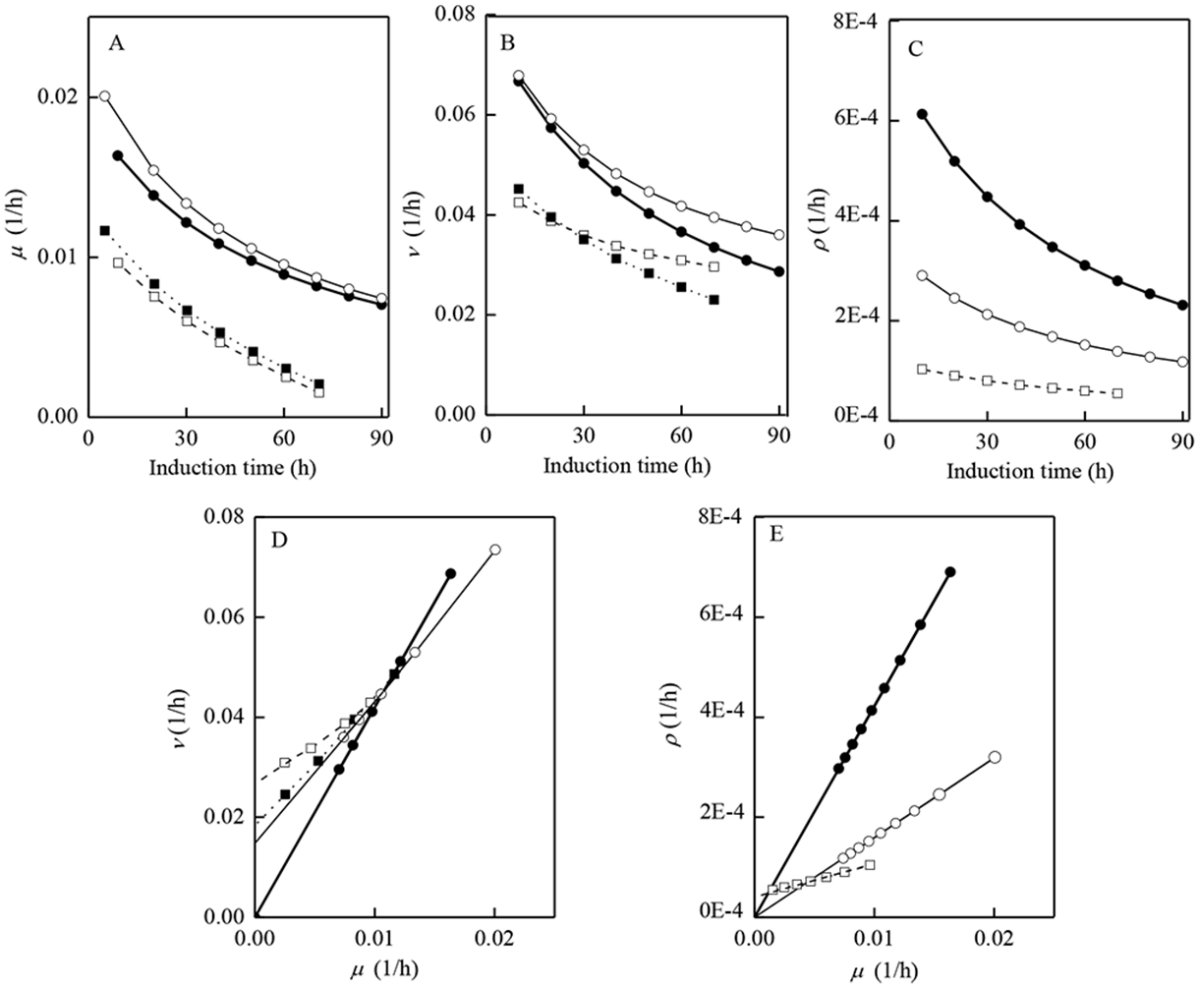
Methanol metabolism patterns under different induction conditions.

The carbon metabolism model and the relevant parameters could then be developed and determined. Fig 4 showed the curves/patterns/values of *μ*, *ν*, *ρ*, “apparent” *Y*_X/S_, *m*, *α* and *β* under different induction strategies (runs #1–3, #6). Table 2 also summarized the major secondary fermentation parameters of *Y*_X/S_ (real *Y*_X/S_), *m*_XE_, *ε*_P_, *γ*, *α* and *β* in different runs (runs #1–8). These results indicated that, compared with the strategy of initiating methanol induction at higher cells concentration, with the strategy of initiating methanol induction at lower cells concentration, 1) much higher specific cells growth rate *μ* and lower cells yield *Y*_X/S_ (larger slope of 1/*Y*_X/S_) were obtained at 30°C/20°C; 2) monellin synthesis was fully associated with cells growth with the highest associated coefficient *α* achieved at 30°C, so that the highest monellin concentration could be obtained; 3) methanol consumption ratio *ε*_P_ in support NADH formation in pass A was higher (22.7%∼29.2% versus 14.0%∼19.0%, runs #1–4 and #6), so that the energy regeneration ability within the entire metabolism was largely enhanced; 4) methanol utilization ratio directed into monellin synthesis route *γ* at 30°C was the largest (65.2%, run #3), allowing sufficient precursors (amino acids, etc.) to participate monellin synthesis. All of the above features/factors greatly contributed the monellin production, particularly for the cases of initiating induction at low cells concentration and 30°C (runs #3–4). There would be some doubt regarding on the high methanol utilization ratio for monellin synthesis *γ* (11.5%∼65.2%, Table 2), since the maximum monellin concentration only stayed at 2.62∼2.71 g/L. It must be addressed that in most cases of foreign proteins production by *P*. *pastoris*, protein concentrations generally stay at single-digital g/L or even mg/L levels, because the expression efficiency is generally low due to mismatch of translation/expression levels. In addition, it should be noted that the Luedeking-Piret model has the universal ability in correlating *μ* (specific cells growth rate) and *ρ* (specific protein synthesis rate) in other runs (run #4, runs #7–8) and the results were very well. This model could also be extended to human serum albumin-fibroblast growth factor 21 fusion protein (HSA-FGF21) production by a Mut^+^ *P*. *pastoris* strain as well (induction at lower cells concentrations and 30°C, unpublished data), indicating its universal ability for modeling protein production by other *P*. *pastoris* strains. Finally, the data in run #1 could not be fitted with Luedeking-Piret model and not shown in Fig 4 because monellin was hydrolyzed immediately after the induction (Fig 2C).

To further investigate the mechanism of the strategy of initiating induction at low cells concentration and to achieve a stable/long term monellin production and increase its productivity, a chemostat fermentation was conducted by initiating induction at about 50 g-DCW/L and 30°C, and then controlling cells concentration around 55 g-DCW/L to maintain the specific cells growth rate (*μ*) at higher level (*D* = *μ* = 0.016 h^-1^). The data of run #3 were used as the reference in determining *D*. In this case, the cells concentration could not be controlled at its desired level, and after starting the chemostat operation, it gradually increased to the level of 80 g-DCW/L in the first 20 h. Then, it roughly maintained around 80 g-DCW/L within the period of 30∼70 h. The big deviations from the desired cells concentration control level might originate from dynamic behavior changes in different fermentation batches, since almost all fermentation processes are characterized with dynamic parameter variations. With regards to monellin concentration in the stirred tank, it could not reach a constant level also. Until the last 20 h in induction phase, monellin concentration seemed to converge to a constant level. Generally, chemostat operation should be lasted for a longer time to indicate its power (productivity increase and long-term/stable production). However, as the metabolic activity began to decline (reflected by OUR, CER, etc.) in the last 20 h, we had to finish the continuous fermentation around 90 h.

Under the chemostat condition (d*X*/dt = 0 & *μ* = *D*), the estimated monellin productivity (*r*_P_) and monellin concentration (*P*) could be calculated as follow:

$$\begin{array}{ll} r_{p}(t) & =\alpha D\bar{X}=\alpha \mu \bar{X}\\ P & =\frac{P_{tf}V+\left(\int_{0}^{tf}r_{p}(t)dt\right)V^{*}}{V+V^{*}}=\frac{P_{tf}V+\left(\int_{0}^{tf}\left(\alpha \mu \bar{X}\right)dt\right)V^{*}}{V+V^{*}} \end{array}$$ (7)

here, *μ*, *F*, *D*, *V*, *α*, *$\bar{X}$*, *P*_tf_, V* and *tf* referred to specific cells growth rate (h^-1^), broth withdrawing/methanol feeding rate (L/h), dilution rate (h^-1^), broth volume in the tank (L), growth-associated coefficient (-), cells concentration at steady-state (g/L), final monellin concentration in the tank (g/L), total broth withdrawing/methanol feeding volume (L) and total induction time, respectively.

We summarized the fermentation performance of the eight (8) different runs, including the chemostat one (run #5) in Table 1. The results indicated that run #3 & #4 (induction at lower cells concentration and 30°C, fed-batch mode) yielded the highest monellin concentration (2.62∼2.71 g/L) and relatively higher productivity (0.029∼0.030 g/L/h). Run #5 (chemostat mode, at 30°C) could obtain the highest productivity (0.044 g/L/h) and relatively higher concentration (2.16 g/L). It must be addressed that, the fermentation index in run #5 were estimated values, because *α* (0.0365) obtained in run #3 was used to calculate *r*_P_ and *P* in Eq 7, though all the other parameters were the real measured one. In the chemostat fermentation (run #5), parameters of *α* and *β* in Luedeking-Piret model are difficult to be identified, as *ρ* (specific product-synthesis rate) and *μ* (specific growth rate) only varied in a very narrow range. However, the features of the higher productivity and concentration of monellin still indicated the potentials of continuous monellin fermentation in the future applications.

### Energy metabolism patterns in monellin production by *P*. *pastoris* under different induction strategies

Energy metabolism patterns or its utilization efficiency is another important issue for the enhanced monellin production by *P*. *pastoris*. Carbon and energy metabolisms must match up with each other to achieve an enhanced fermentation performance. As shown in Table 2, methanol distribution ratio towards into precursors synthesis route seemed to be reasonably higher when initiating induction at lower cells concentration and 30°C but did not increase such much (*γ* = 47.8%, run#1; *γ* = 51.9∼65.2%, run #3–4; Table 2). However, the methanol ratio towards into energy metabolism in supporting monellin synthesis/cells growth increased largely (*ε*_P_ = 14.0%, run#1; *ε*_P_ = 22.7∼27.2%, run #3–4; Table 2). The methanol/energy metabolisms ratio in run #3–4 seemed to be regulated at a more balanced level. In addition, energy utilization efficiency is extremely important in *P*. *pastoris* metabolism and it strongly relies on oxygen uptake and ATP regeneration rates in oxidative phosphorylation reaction. As shown in Fig 3, oxygen utilization is involved with two steps within the overall methanol metabolism. The first step is the formaldehyde (HCHO) oxidation from methanol catalyzed by AOX, which is the central and first metabolic reaction in methanol metabolism. The O_2_ uptake rate in this step *r*_O2_^(1)^ could be (on-line) determined by methanol consumption rate *r*_MeOH_ and the following reaction stoichiometric coefficient.

$$CH_{3}OH(MeOH)+\frac{1}{2}O_{2}\overset{AOX}{\to }CH_{2}O+H_{2}O$$ (8)

$$r_{O2}^{\left(1\right)}(T,t)=\frac{1}{2}r_{MeOH}(T,t)$$ (9)

Here, *T* and *t* represented temperature and induction time, respectively. The second step is the oxidative phosphorylation reaction where ATP was regenerated from NADH formed in pathway A by consuming another portion of oxygen, and the O_2_ uptake rate in this step *r*_O2_^(2)^ could be determined by using the measured OUR and Eq 11.

$$\frac{1}{2}O_{2}+NADH+(P/O)ADP\overset{Pi}{\to }(P/O)ATP+NAD^{+}+H_{2}O$$ (10)

$$r_{O2}^{\left(2\right)}(T,t)=OUR(T,t)-r_{O2}^{\left(1\right)}(T,t)=OUR(T,t)-\frac{1}{2}r_{MeOH}(T,t)$$ (11)

$$r_{NADH}^{F}(T,t)=2\;CER(T,t)$$ (12)

$$r_{NADH}^{C}(T,t)=2\;r_{O2}^{\left(2\right)}(T,t)$$ (13)

$$r_{ATP}(T,t)=(P/O)r_{O2}^{\left(2\right)}=(P/O)r_{NADH}^{C}(T,t)$$ (14)

Here, *r*_MeOH_, *r*^F^_NADH_, *r*^C^_NADH_, *r*_ATP_, OUR and CER represented the rates of methanol consumption, NADH formation, NADH consumption, ATP regeneration in the oxidative phosphorylation reaction, O_2_ uptake and CO_2_ evolution.

Theoretically, ATP regeneration rate *r*_ATP_ depends on *r*_O2_^(2)^ and *r*^C^_NADH_ as described by Eq 14. Assuming P/O ratio under different induction temperatures is identical, then ATP regeneration rate *r*_ATP_ could be described as *r*_ATP_ = (P/O) *r*^C^_NADH_. Here, we defined a new parameter *η* to represent the energy utilization efficiency.

$$\eta =\frac{r_{NADH}^{C}}{r_{NADH}^{F}}$$ (15)

**Fig 5:**
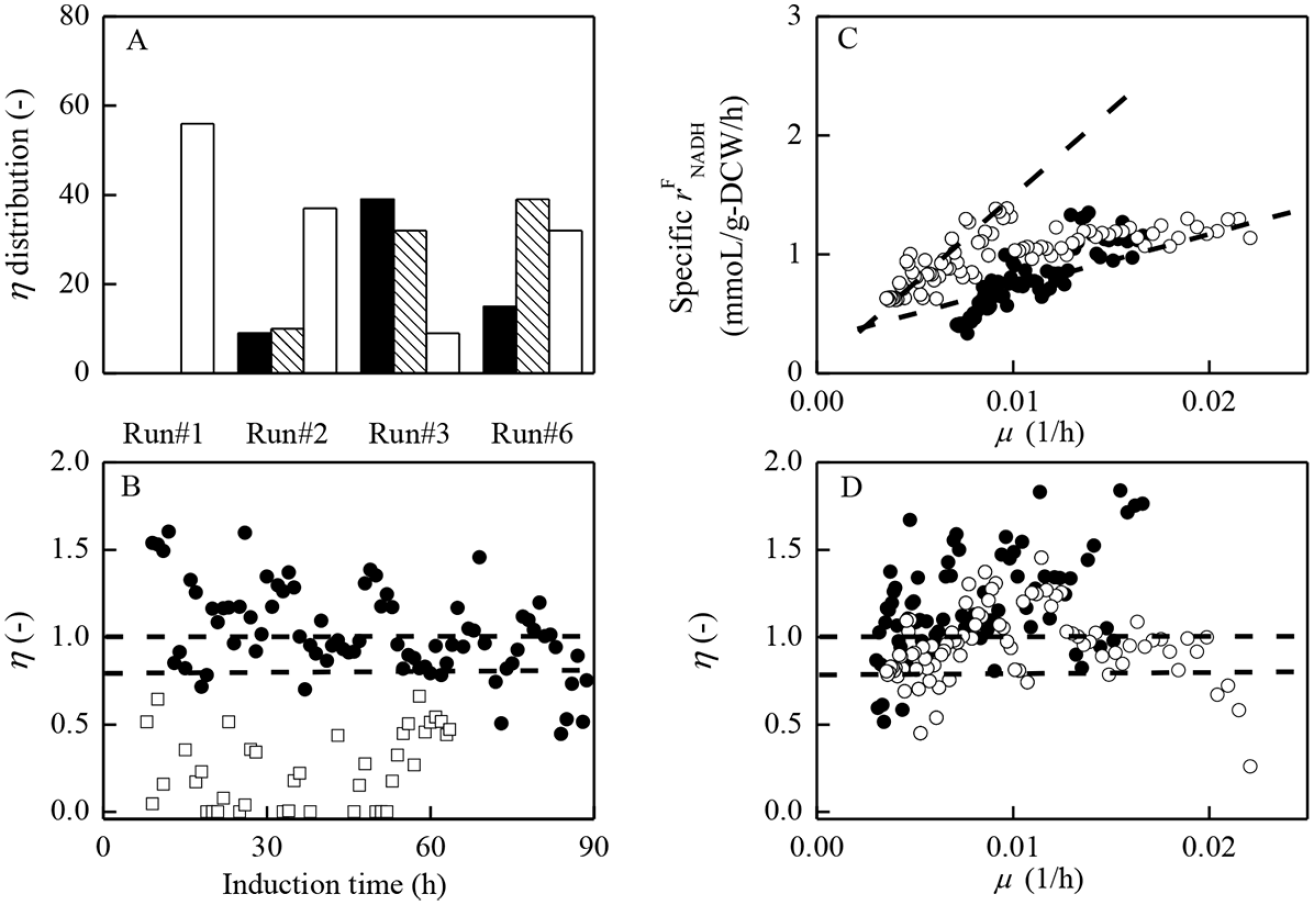
Energy (NADH) metabolism patterns *η* under different induction strategies.

*η* (*r*^C^_NADH_/*r*^F^_NADH_) could be recognized as the energy (NADH) utilization efficiency for heterologous protein production by *P*. *pastoris*. Theoretically, *η* = 1 implies that, all of the formed NADH could be fully utilized in the oxidative phosphorylation reaction required for heterologous proteins synthesis and cells growth as shown in Fig 3. If *η* >1, NADH requirement amount exceeds the NADH amount formed, which means that NADH consumption amount is limited, and the maximum *r*^C^_NADH_ would have to equal *r*^F^_NADH_. We categorized the energy (NADH) utilization efficiency as follows: If *η*≥1, NADH generated in Path A could be fully (100%) utilized to form ATP supporting monellin synthesis and cells growth; if 0.8<*η*<1.0, the energy utilization efficiency is at moderately high level; while if *η*≤0.8, then the energy utilization efficiency is considered to be at low level, the NADH formed could not be effectively utilized for ATP regeneration in support of monellin synthesis and cells growth. Fig 5A and Table 2 depicted the categories of *η* within one hour sampling interval during the entire induction phase for runs #1–3 and #6. As shown in Fig 5A and Table 2, the ratio of data categorized into high *η* (*η*≥1, very high; 0.8<*η*<1.0 moderately high) in run #3 (initiating induction at lower cells concentration and 30°C) was the highest (*η*≥1, 49%; 0.8<*η*<1.0, 40%; a total of 89%. The 0.8<*η*<1.0 plus *η*≥1.0 ratio in run #4 also reached higher level of 79%), while that categorized into low *η* in run #1 (initiating induction at higher cells concentration and 30°C) was largest (*η*≤0.8, low) with a percentage of 100% (*η*≤0.8, 100%; 0.8<*η*<1.0 plus *η*≥1.0, 0%). The ratios of data categorized into high *η* (0.8<*η*<1.0 plus *η*≥1.0) in run #2 (34%) and #6 (63%) were in the middle of those of run #1 and run #3. Fig 5B showed time variation of *η* distribution for the two extreme runs (run #1 and #3).

As shown in Fig 2 and Table 1, the monellin concentration in run #1 was the lowest and monellin hydrolyzed immediately after the induction. We interpreted the results as follows: 1) the energy/methanol metabolisms ratio in run #1 was at an un-balanced state (*ε*_P_/*γ* = 0.29, run #1 versus *ε*_P_/*γ* = 0.35, run #3); 2) 100% of *η* data in run #1 were under 0.8 (*η*≤0.8, Fig 5B), and the very low energy (NADH) utilization efficiency *η* could be considered as another major reason responsible for the lowest monellin concentration of 0.54 g/L. On the contrast, most of *η* data (89%) in run #3 were above 0.8 (*η*>0.8), suggesting that NADH formed in pass A could be effectively utilized for ATP regeneration, leading to the higher specific cells growth rate (*μ*, Fig 4A) and methanol utilization ratio for monellin synthesis (*γ*, Table 2), and eventually the highest monellin concentration of 2.62∼2.71 g/L was achieved in run #3–4. We must address that data of *η*>1 is not realistic (*η* = 1 is the theoretically maximum value), and this discrepancy may occur from the determination of OUR, CER and methanol consumption rate (Eqs 11–13). Since those on-line measurement systems were independent, and OUR/CER were sensitive to the frequent air drying agents (silica) exchange (to protect analysis chamber of the gas analyzer) and the electronic balance suffered with a “negative” measurement deviation (its reading automatically decreased about 10∼20 g per 10 h even without methanol feeding). As many of *η* data above 1 were below 1.00∼1.25, we believed this small deviation can be ascribed to the serious measurement noise common in fermentation processes. However, none of *η* data were above 0.8 in run #1. These data could at least be used in interpreting the large differences in NADH utilization efficiency and monellin concentrations in the two extreme runs.

### Analysis of gene transcriptional levels of key enzymes and enzymatic activities in carbon/energy metabolic pathways in monellin fermentation process using different induction strategies

**Fig 6:**
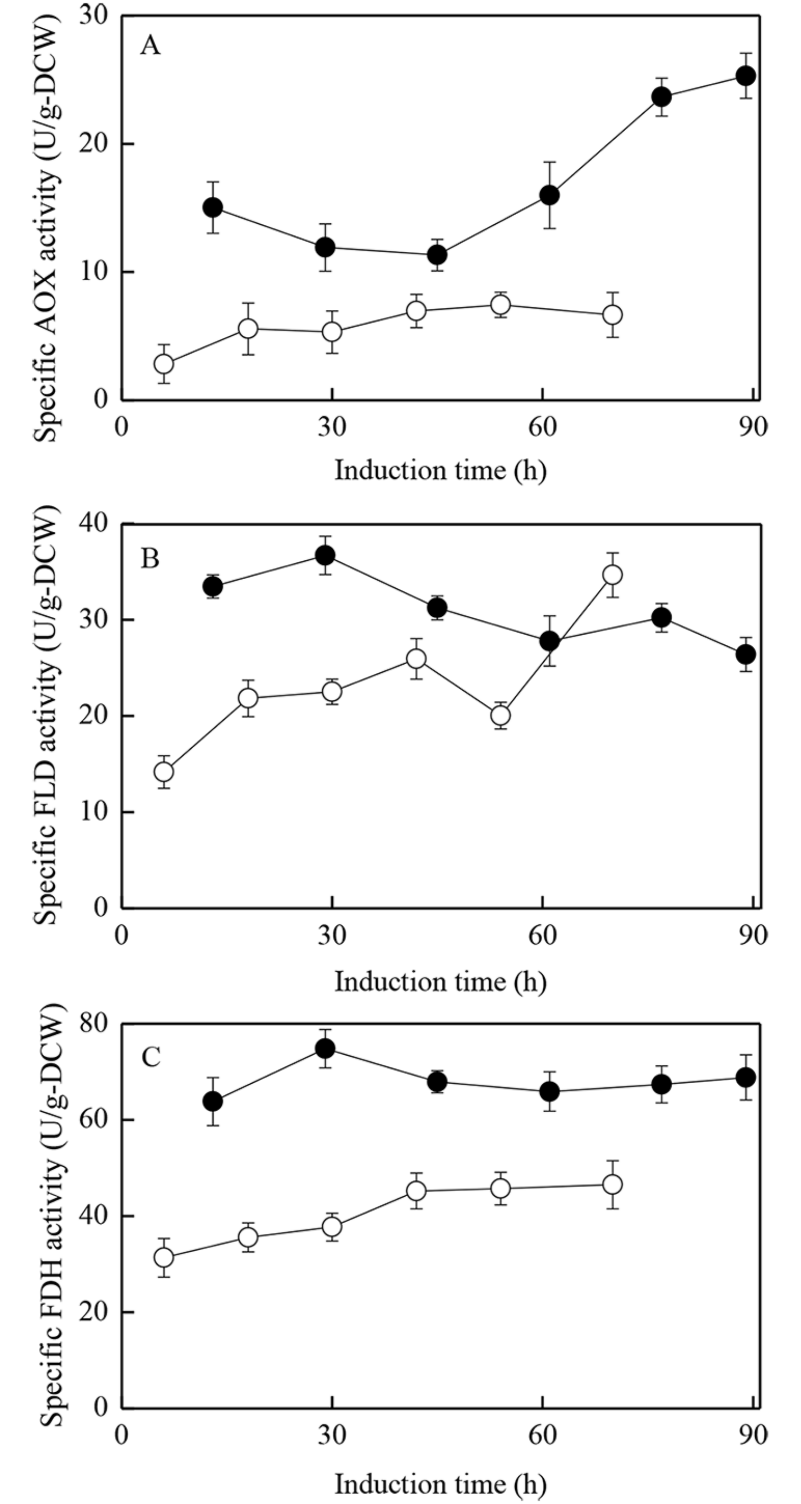
Specific activities of AOX, FLD, and FDH in run #1 and #3.

The gene transcriptional levels of key enzymes (at 36 h after initiating induction) in carbon/energy metabolic pathways in the monellin fermentation process were measured in supporting the carbon/energy metabolism patterns analysis. The transcriptional levels of four key genes, *aox2*, *fld1*, *fdh1* and *das1* in the two extreme cases (run #1 and #3, maximum monellin concentrations of 0.54 g/L and 2.62 g/L) were analyzed and summarized in Table 3. As shown in Fig 3, *aox2* is the gene encoding alcohol oxidase (AOX) which dominates the entire carbon metabolism; *fdh1* and *fld1* are the genes encoding formaldehyde dehydrogenase (FLD) and formate dehydrogenase (FDH) which contribute to NADH regeneration ability and detoxification of formaldehyde/formate in the dissimilation (energy) pathway (pass A); *das1* is the gene encoding dihydroxyacetone synthase (DAS) which could enhance methanol utilization rate in the assimilation pathway (cells growth/maintenance & precursors synthesis) and repress the toxicity of H_2_O_2_ in peroxisome (pass B). As shown in Table 3, all the transcriptional levels of *aox2*, *fld1*, *fdh1* and *das1* were significantly up-regulated in run #3 compared with those of run #1. These results totally agreed with and supported the conclusions on the carbon/energy metabolism patterns in run #1 and #3. In addition, the enzymatic activities of AOX, FLD, and FDH in the two extreme cases (run #1 & #3) were also measured and depicted in Fig 6. As shown in Fig 6, the enzymatic activities of the three (3) enzymes were largely enhanced in run #3, as compared with those of run #1.

**Table 3:** Transcription levels of key genes in methanol metabolism of monellin fermentation process.

	Gene	Gene function	Regulated	Transcriptional level
Run #1	*aox2*	Alcohol oxidase2	-	4.1
	*fld1*	Formaldehyde dehydrogenase	-	1.4
	*fdh1*	Formate Dehydrogenase	-	36.5
	*das1*	Dihydroxyacetone synthase	-	6.8
Run #3	*aox2*	Alcohol oxidase2	UP	9.6
	*fld1*	Formaldehyde dehydrogenase	UP	2.0
	*fdh1*	Formate Dehydrogenase	UP	84.4
	*das1*	Dihydroxyacetone synthase	UP	12.9

### Effects of temperature and DO on monellin production and energy utilization efficiency

Besides the cells concentration when initiating methanol induction, induction temperature and DO level during induction phase are the other crucial factors for the heterologous protein productions by *P*. *pastoris*. It has been reported that low induction temperature at 20°C could enhance AOX activity [29], decrease hydrolysis of the targeted proteins [17], increase the energy regeneration efficiency [11], and further enhance production (concentration) of the targeted proteins [11, 17, 29]. In monellin production by *P*. *pastoris*, some of the above conclusions were consistent with the results (such as AOX, etc., data not shown) but some others (NADH utilization efficiency *η*, monellin concentration & synthesis rate *ρ*, etc.; Figs 2, 4 & 5, Tables 1 and 2) were not. As shown in Fig 3, if AOX was activated by reducing the induction temperature, then a large amount of O_2_ (*r*_O2_^(1)^) would be consumed in methanol dissimilation route which greatly increased *r*^F^_NADH_ but decreased O_2_ available for the oxidative phosphorylation reaction *r*_O2_^(2)^ and *r*^C^_NADH_, and thus resulting a significant decrease in *r*_O2_^(2)^, *r*^C^_NADH_ and *η* which may vary rates of cells growth and monellin synthesis in turn. Fig 4 and Table 2 indicated that, when initiating methanol induction at low cells concentration, specific cells growth rate were almost the same but the cells growth associated coefficient *α* (run #3 versus #6, Eq 6, Table 2) was more than doubled at 30°C induction compared with that at 20°C (Fig 4). On the other hand, as shown in Fig 5C and 5D, the associated pattern of specific *r*^F^_NADH_ versus specific cells growth rate (*μ*) basically followed same patterns (Eq 16) for different induction temperature (20°C/30°C), but energy utilization efficiency *η* decreased when inducing at 20°C particularly in higher specific cells growth rate (*μ*) region (Fig 5D).

$$\frac{r_{NADH}^{F}}{X}=\frac{1}{Y_{NADH}}\mu +m_{NADH}$$ (16)

Here, *X* referred to cells concentration. As a result, we concluded that initiating methanol induction at lower cells concentration while maintaining induction temperature at 30°C is the sub-optimal strategy. This strategy could not only increase monellin synthesis rate and NADH utilization efficiency *η*, but also relieve the heat exchange/oxygen supply loads/costs particularly in the summer season.

DO levels is also a crucial issue for heterologous protein productions by *P*. *pastoris*. It is reported that the higher DO during induction phase was beneficial for heterologous protein production [30]. However, this was not the case in this study. The results (Table 1 and Fig 2) indicated that maintaining DO at higher levels (10–60%, induction at 20°C/30°C) by aerating pure oxygen with the strategy of initiating induction at low cells concentration could not further increase monellin productions.

## Conclusions

In monellin production by *P*. *pastoris*, the strategy of initiating induction at lower cells concentration and 30°C was found to be best fermentation mode yielding the highest monellin concentration. We fulfilled theoretical analysis of methanol/energy metabolisms for this fermentation and compared the results with other induction strategies. The results revealed that enhanced monellin production in this case was due to the following: sufficient methanol distribution fluxes available in precursors synthesis and NADH formation routes; high growth associated coefficient *α* and specific growth rate; highest NADH utilization efficiency allowing NADH to be effectively utilized in supporting cells growth and monellin synthesis. Furthermore, this strategy could reduce monellin production costs by relieving the requirement on heat exchange and pure oxygen supply load.

## Supporting information

S1 File (PDF)

## Abbreviations

*A*^T^MeOH: total methanol consumption amount (mmol/L or g/L)
CER: CO_2_ evolution rate (mmol/L/h)
*D*: dilution rate (h^-1^)
DCW: dry cell weight (g-DCW/L or g/L)
DO: dissolved oxygen concentration (%)
*F*: broth withdrawing/methanol feeding rate (L/h)
*k*: the slope of *μ/ν* versus 1/*ν* (-, Eq 4)
*m*: “apparent” cells maintenance yield on methanol (h^-1^, Eq 1)
*m*_NADH_: NADH consumption rate for maintenance (mmol/g-DCW/h, Eq 16)
*m*_XE_: pure methanol utilization ratio for cells maintenance (-, Eq 5)
OUR: O_2_ uptake rate (mmol/L/h)
*P*: estimated monellin concentration in run #5 (g/L)
*P*_tf_: final monellin concentration in the tank in run #5 (g/L)
*r*_ATP_: ATP regeneration rate (mmol/L/h)
*r*^C^NADH: NADH consumption rate (mmol/L/h)
*r*^F^NADH: NADH formation rate (mmol/L/h)
*r*_MeOH_: methanol consumption rate (mmol/L/h)
*r*_O2_^(1)^: O_2_ uptake rate in formaldehyde oxidation from methanol (mmol/L/h)
*r*_O2_^(2)^: O_2_ uptake rate in oxidative phosphorylation reaction (mmol/L/h)
*r*_P_: monellin productivity (g/L/h)
*t*_f_: total induction time (h, Eq 7)
*T*: total induction time (h, Eq 5)
Δ*T*: sampling interval (h, Eq 5)
*V*: broth volume in the tank (L)
*V**: total broth withdrawing/methanol feeding volume (L, Eq 7)
*X*: cells concentration (g/L)
$\bar{X}$: cells concentration at steady-state condition in Eq 7 (g/L)
*Y*_NADH_: cells yield on NADH (g-DCW/mmol, Eq 16)
*Y*_X/S_: “apparent” cells yield on methanol (-, Eq 1)
*α*: the associated coefficient in correlating specific cells growth rate (*μ*) and specific monellin synthesis rate (*ρ*) (-, Eq 6)
*β*: the non-associated coefficient in correlating specific cells growth rate (*μ*) and specific monellin synthesis rate (*ρ*) (-, Eq 6)
*γ*: methanol utilization ratio for monellin synthesis/cells growth (-)
*ε*: carbon distribution ratio for the total energy (NADH) for monellin synthesis, cells growth and cells mainteinance (-)
*ε*_P_: carbon distribution ratio for the energy (NADH) for monellin synthesis and cells growth route (-)
*η*: energy (NADH) utilization efficiency (-)
*μ*: specific cells growth rate (h^-1^)
*v*: specific methanol consumption rate (h^-1^)
*ρ*: specific monellin synthesis rate (h^-1^)
