# Correction: Enhancing monellin production by *Pichia pastoris* at low cell induction concentration via effectively regulating methanol metabolism patterns and energy utilization efficiency

**DOI:** 10.1371/journal.pone.0201085

**Published:** 2018-07-18

**Authors:** Luqiang Jia, Tingyong Tu, Qiangqiang Huai, Jiaowen Sun, Shanshan Chen, Xin Li, Zhongping Shi, Jian Ding

The captions for Fig 1 through Fig 6 are missing. Please see the complete captions here:

**Fig 1:**
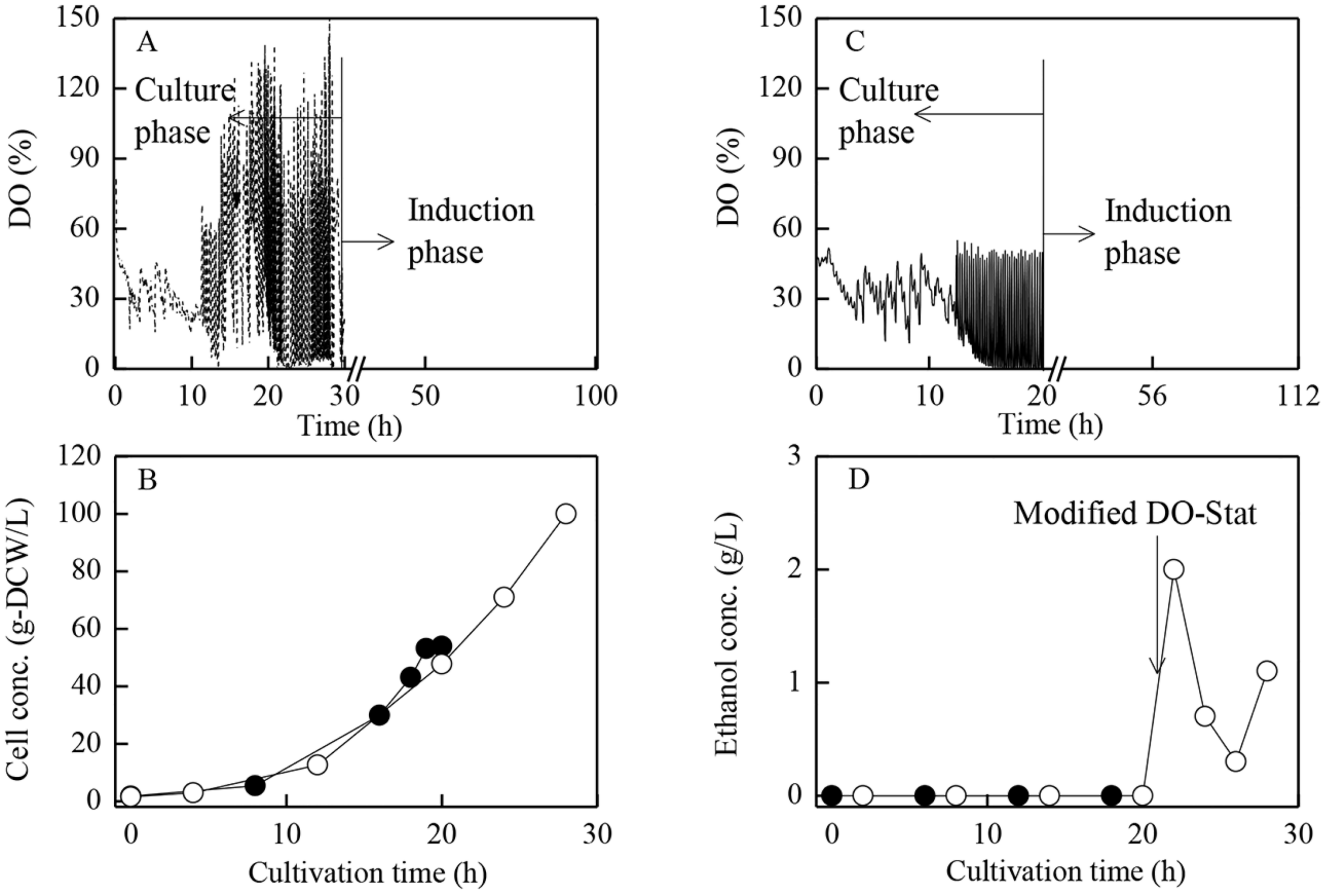
Time courses of DO, cell and ethanol concentrations during cells growth phase under different strategies. ○: by standard strategy initiating induction at high cells concentration with pure oxygen aeration at 14 h and implementing the modified glycerol feeding strategy at about 22 h (A). ●: initiating induction at low cells concentration with air aeration/standard DO-Stat feeding (C).

**Fig 2:**
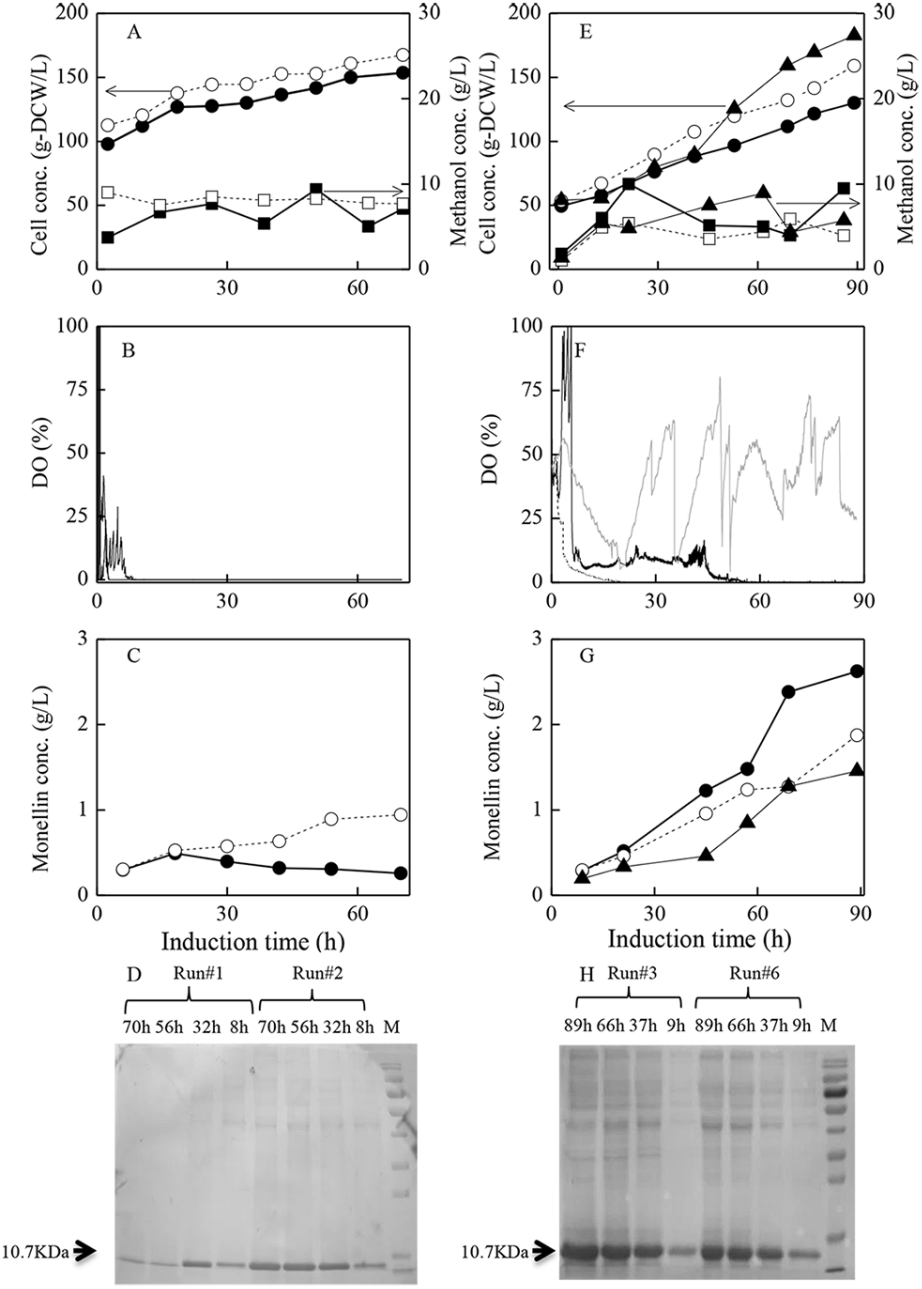
Curves of fermentation data and monellin synthesis/SDS analysis results during induction phase with different strategies. (A)-(D): initiating methanol induction at high cell concentration; (E)-(H): initiating induction at low cell concentration. ●, ■ and □: induction at 30°C; ○, □ and ┄: at 20 °C; ▲ and —: at 30 °C but aerating pure oxygen.

**Fig 3:**
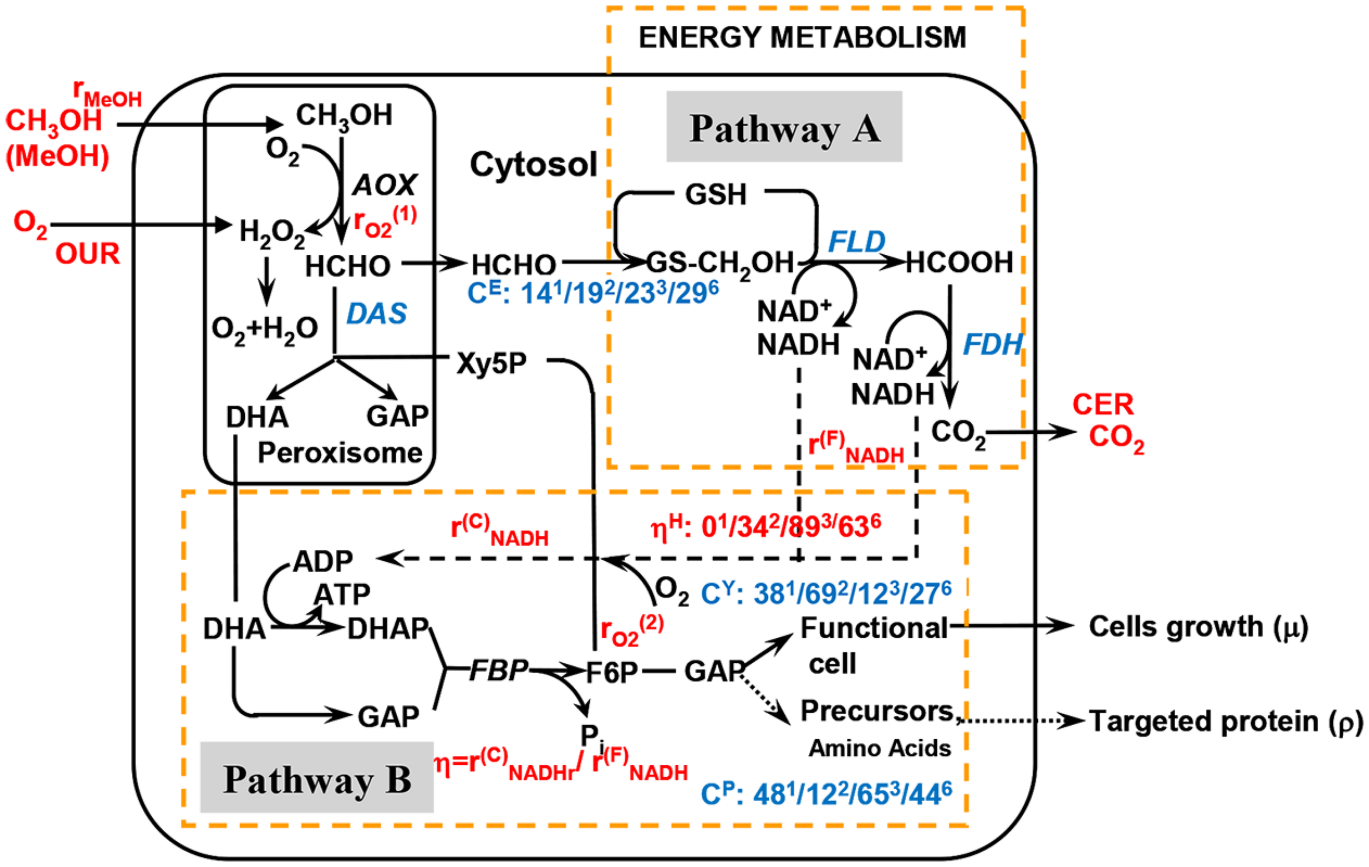
A simplified carbon/metabolic map and the carbon/energy distribution ratios for monellin synthesis by *P*. *pastoris*. C^Y^: methanol distribution ratio for cells growth and maintenance; C^P^: methanol distribution ratio for protein synthesis; C^E^: methanol distribution ratio for NADH (energy) formation. *η*^H^: the ratio of data categorized into high energy utilization efficiency *η* (*η*>0.8). 1, 2, 3 and 6: referred to fermentation runs numbers.

**Fig 4:**
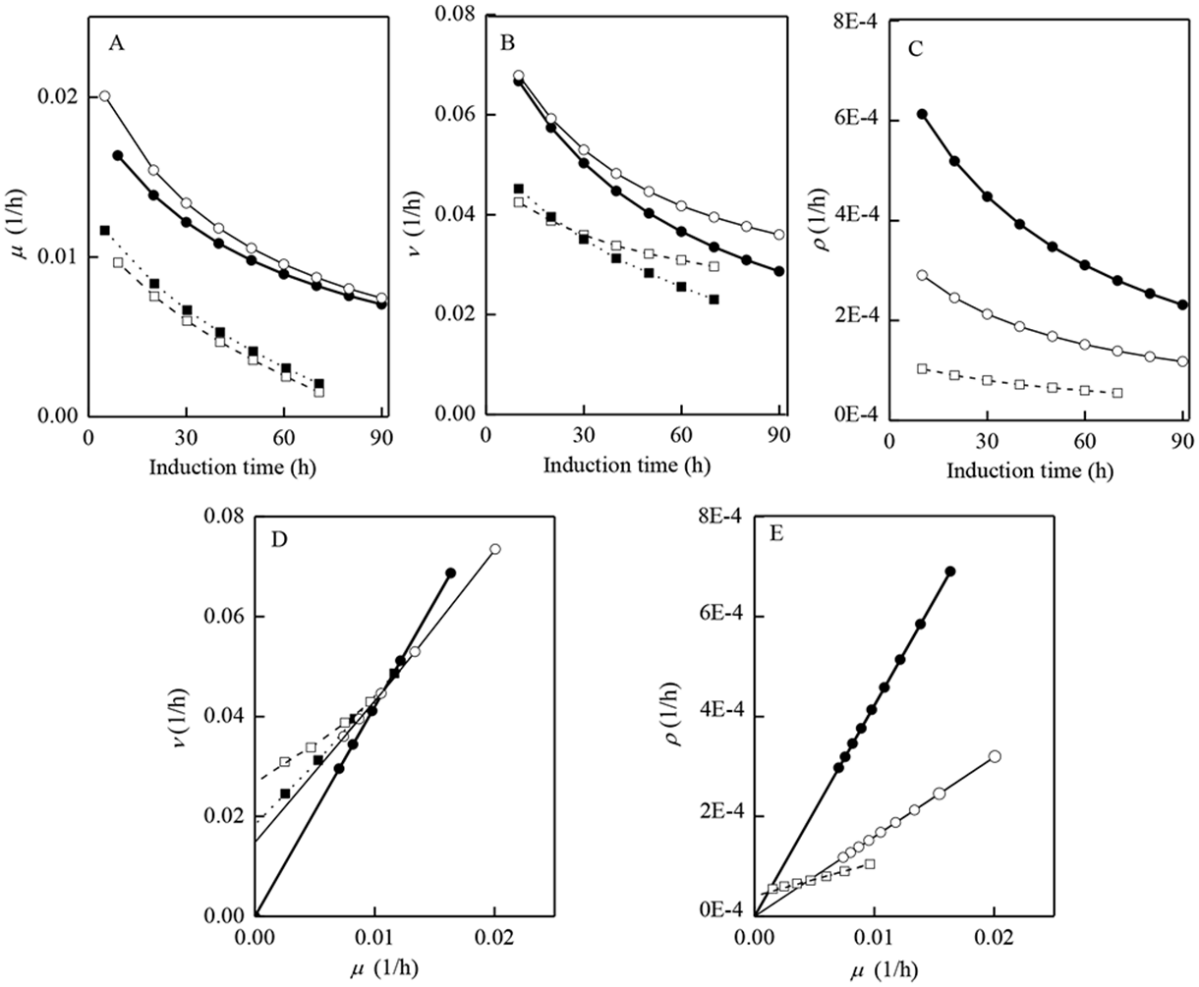
Methanol metabolism patterns under different induction conditions. (A): specific cells growth rates (*μ*); (B): specific methanol consumption rates (*v*); (C): specific monellin production rates (*ρ*); (D): specific methanol consumption rates (*v*) versus specific cells growth rates (*μ*); (E): specific monellin production rates (*ρ*) versus specific cells growth rates (*μ*). ● and □: induction at low cells concentration and 30°C (run #3); ○ and —: at low cells concentration and 20°C (run # 6); ■ and …: at high cells concentration and 30°C (run #1); □ and ┄: at high cells concentration and 20°C (run #2).

**Fig 5:**
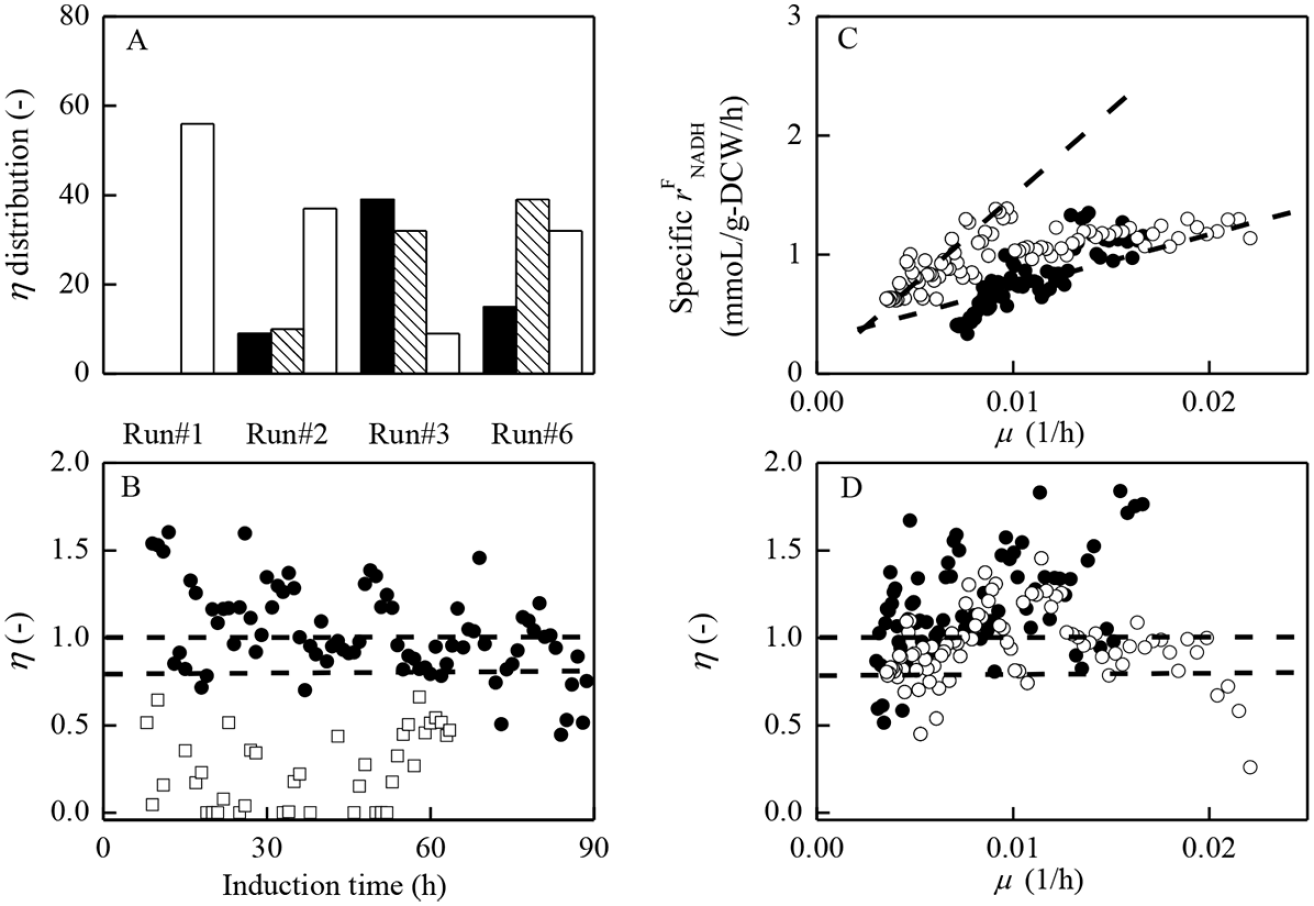
Energy (NADH) metabolism patterns □ under different induction strategies. (A): Categories of NADH distribution *η* (*r*^C^_NADH_/*r*^F^_NADH_) under different induction strategies (runs #1–3, #6). █: *η*≥1.0; ░: 0.8<η<1.0; □: 0.0≤η≤0.8. (B): NADH distribution *η* (*r*^C^_NADH_/r^F^_NADH_) versus induction time under the two extreme induction strategies (run #1 and #3). □: run #1; ●: run #3. (C): specific *r*^F^_NADH_ versus specific growth rate (*μ*) when initiating induction at low cell concentration while maintaining induction temperature at 30°C/20°C. ●: run #3, ○: run #6. (D): NADH distribution *η* (*r*^C^_NADH_/*r*^F^_NADH_) versus specific growth rate (*μ*) when initiating induction at low cell concentration while maintaining induction temperature at 30°C/20°C. ●: run #3, ○: run #6.

**Fig 6:**
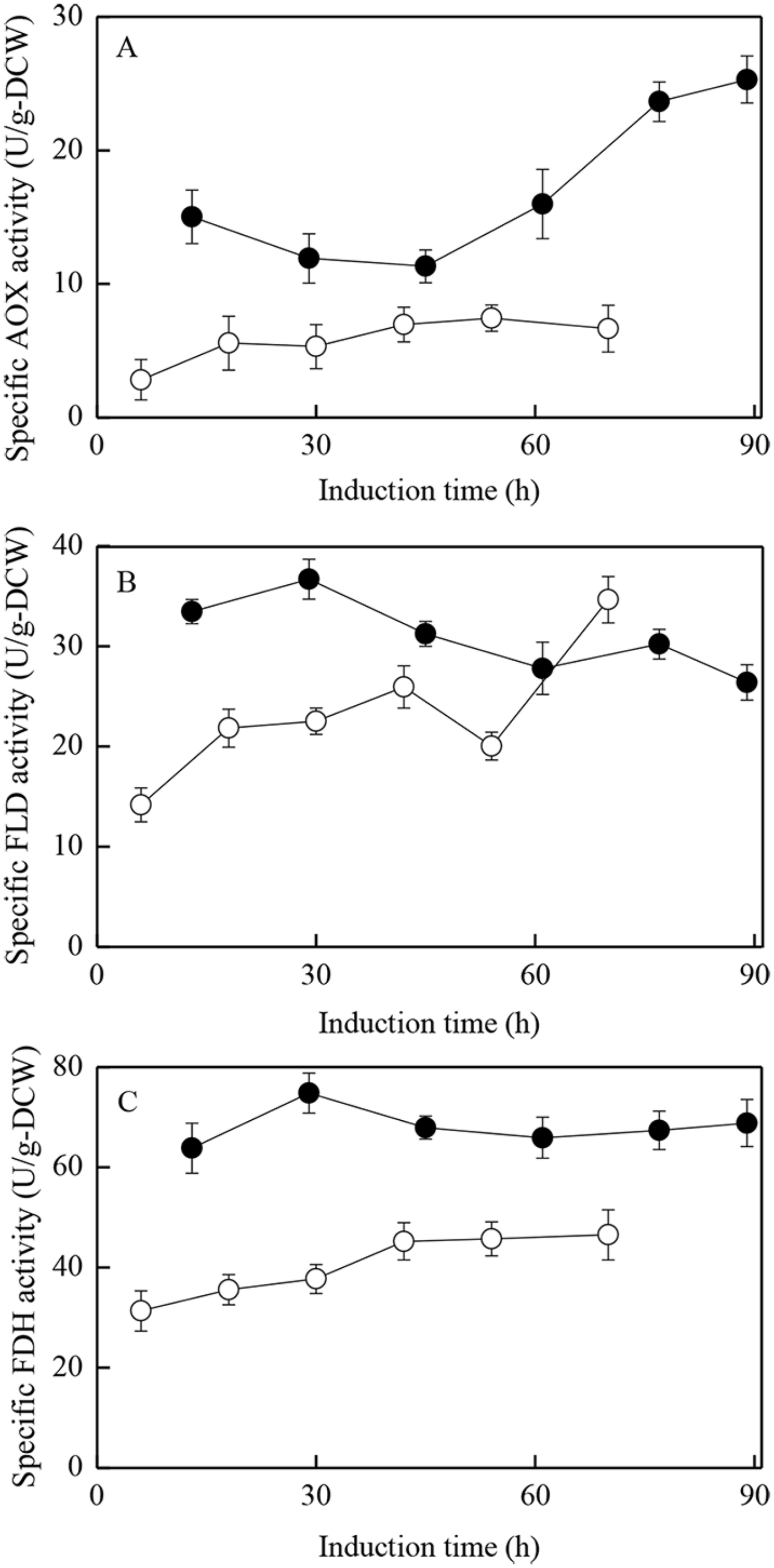
Specific activities of AOX, FLD, and FDH in run #1 and #3. ○: run#1; ●: run#3.
