# Supplementary material for: Enhancing monellin production by Pichia pastoris at low cell induction concentration via effectively regulating methanol metabolism patterns and energy utilization efficiency
Source: PLoS One. 2017 Oct 5;12(10):e0184602. doi: 10.1371/journal.pone.0184602 (PMC5628809; doi:10.1371/journal.pone.0184602)
Supplement: S1 File — (PDF) [file pone.0184602.s001.pdf]

# Supporting Information

**Enhancing monellin production by *Pichia pastoris* at low cells induction concentration via effectively regulating methanol metabolism patterns and energy utilization efficiency**

Luqiang Jia[1], Tingyong Tu[1], Qiangqiang Huai[1], Jiaowen Sun[1], Shanshan Chen[1], Xin Li[2], Zhongping Shi[1*], Jian Ding[1**]

[1] The Key Laboratory of Industrial Biotechnology, Ministry of Education, School of Biotechnology, Jiangnan University, Wuxi, Jiangsu, China

[2] School of Biology and Pharmaceutical engineering, Wuhan Polytechnic University, Wuhan, Hubei, China.

[*]Corresponding authors:

Email: zpshi@jiangnan.edu.cn (ZS)

[**]Corresponding authors:

Email: dingjian@jiangnan.edu.cn (JD)

**Table S1 Fermentation performance comparisons of HSA-FGF21 production by *P. pastoris* with different induction strategies.**

| Run# | Induction Temp. (°C) | Initial DCW* (g/L) | Induction time (h) | Final DCW (g/L) | Max protein conc. (mg/L) | Ave. MeOH conc.(g/L) | Ave. DO (%) | Aeration Mode |
|---|---|---|---|---|---|---|---|---|
| A | 30 | 62.5 | 89 | 142.5 | 130 | ~5.0 | ~0 | Air |
| B | 30 | 102.3 | 70 | 149.3 | 40 | ~5.0 | ~0 | Air |

Note:

1) DCW: Dry Cells Weight; MeOH: Methanol.

2) *: Cell concentration when methanol induction was initiated.

**Table S2 Major secondary fermentation parameters in HSA-FGF21 production by *P. pastoris* with different induction strategies.**

| | Carbon Distribution Ratios (%) | | | | HAS-FGF21 Synthesis Parameters | | $\eta$ Distribution (%) | |
|---|---|---|---|---|---|---|---|---|
| Run# | Cells Growth $Y_{X/S}$ | Maintenance $m$ | Energy $\varepsilon$ | Precursors $\gamma$ | $\alpha$ | $\beta$ | $\eta$>0.8 | 0≤$\eta$≤0.8 |
| A | 13.6 | 9.0 | 37.7 | 39.7 | 0.00200 | 0.00000 | 27.4 | 72.6 |
| B | 18.1 | 21.7 | 43.1 | 17.1 | - | - | 0.0 | 100.0 |

**Table S3 Selection of dilution rate *D* for the chemostat monellin production using the parameters obtained in run #3 as the reference base.**

| Induction time (h) | $\mu$ (1/h) | Cell concentration (g/L) | $r_p$ (g/L/h) |
|---|---|---|---|
| 9 | 0.01635 | 53.5 | 0.03193 |
| 21 | 0.01367 | 62.8 | 0.03133 |
| 45 | 0.01029 | 90.0 | 0.03380 |
| 57 | 0.00926 | 97.3 | 0.03289 |
| 69 | 0.00825 | 111.5 | 0.03357 |
| 89 | 0.00710 | 130.0 | 0.03369 |

**Table S4 Comparison of monellin productivity and concentration in different runs.**

| Batch/run no. | $r_p$ (g/L/h) | P (g/L) |
|---|---|---|
| #1 | 0.00771 | 0.54 |
| #2 | 0.01486 | 1.04 |
| #3 | 0.02940 | 2.62 |
| #4 | 0.03040 | 2.71 |
| #5 | 0.04380* | 2.16*/1.02** |
| #6 | 0.02101 | 1.87 |
| #7 | 0.01854 | 1.65 |
| #8 | 0.01640 | 1.46 |

Note: *: estimated value using Eq.7.

**: measured value at the fermentation end.

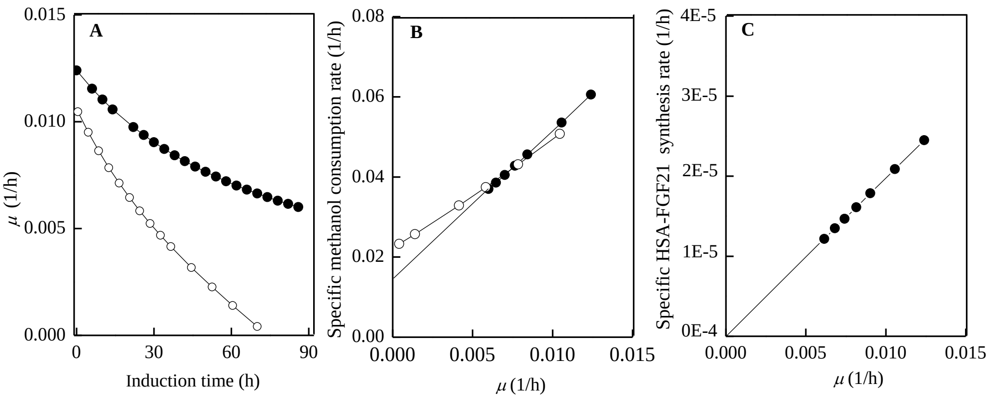
A
0.015
0.010
0.005
0.000
μ (1/h)
0
30
60
90
Induction time (h)
B
0.08
0.06
0.04
0.02
0.00
Specific methanol consumption rate (1/h)
0.000
0.005
0.010
0.015
μ (1/h)
C
4E-5
3E-5
2E-5
1E-5
0E-4
Specific HSA-FGF21 synthesis rate (1/h)
0.000
0.005
0.010
0.015
μ (1/h)

**Fig.S1**

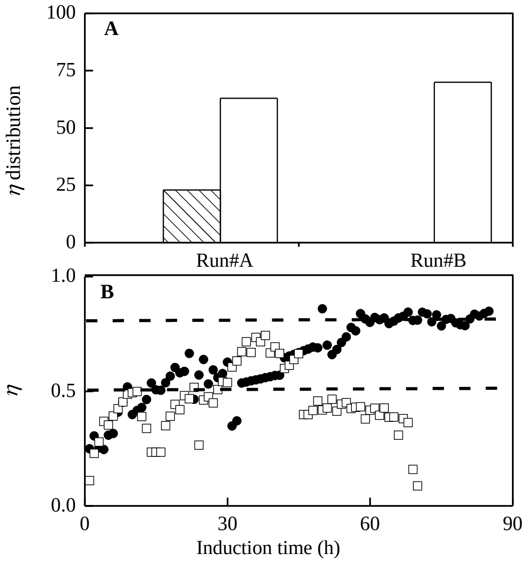
A
100
75
50
25
0
η distribution
Run#A
Run#B
B
1.0
0.5
0.0
η
0
30
60
90
Induction time (h)

**Fig.S2**

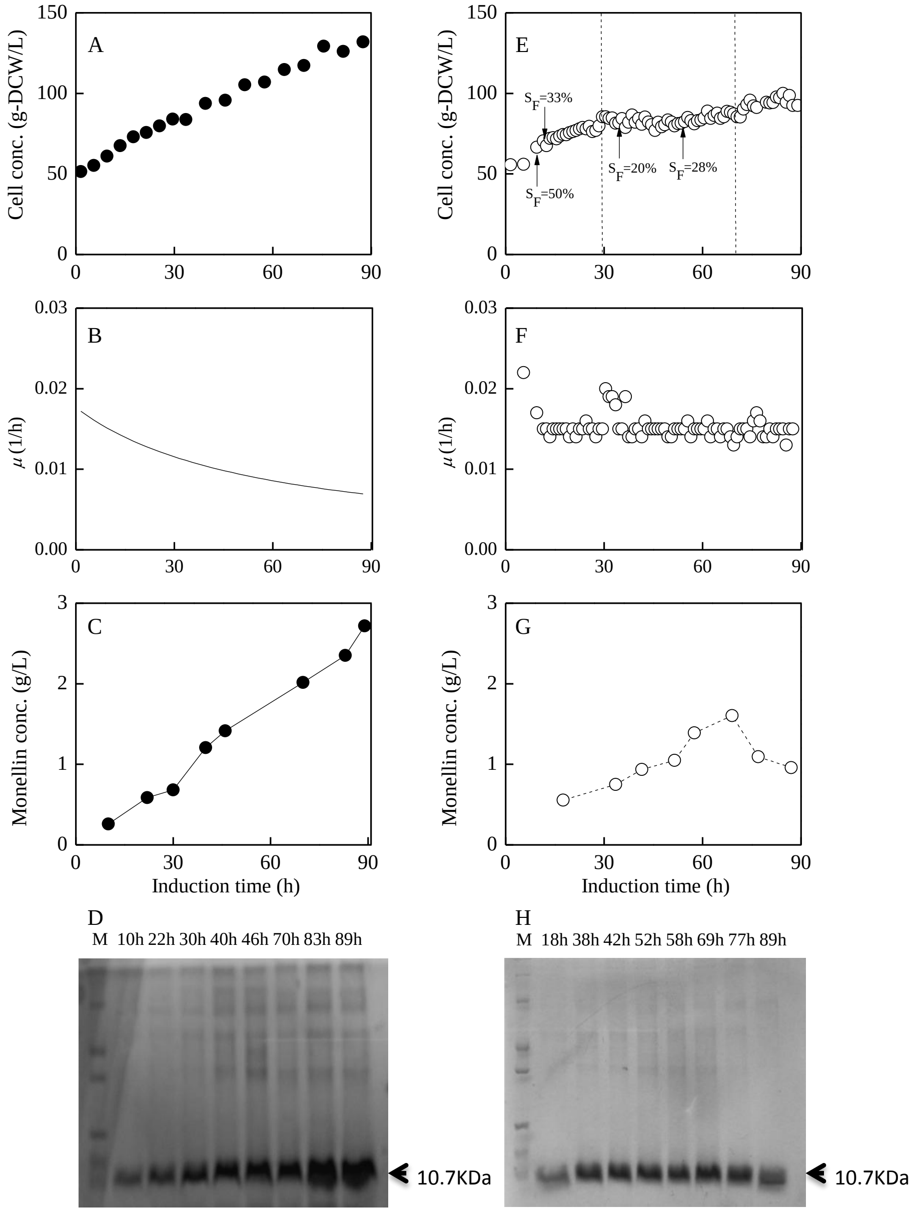
A
B
C
D
E
F
G
H
Cell conc. (g-DCW/L)
μ (1/h)
Monellin conc. (g/L)
Induction time (h)
S_F=33%
S_F=50%
S_F=20%
S_F=28%
M 10h 22h 30h 40h 46h 70h 83h 89h
M 18h 38h 42h 52h 58h 69h 77h 89h
10.7KDa
10.7KDa

**Fig.S3**

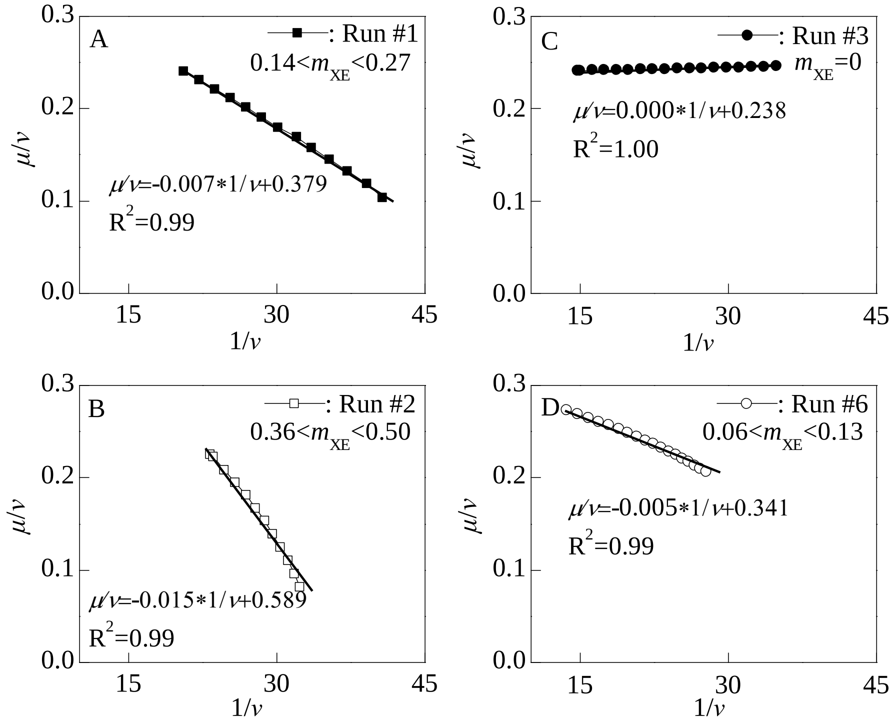

A
: Run #1
0.14<mXE<0.27
μ/ν=-0.007*1/ν+0.379
R2=0.99
C
: Run #3
mXE=0
μ/ν=0.000*1/ν+0.238
R2=1.00
B
: Run #2
0.36<mXE<0.50
μ/ν=-0.015*1/ν+0.589
R2=0.99
D
: Run #6
0.06<mXE<0.13
μ/ν=-0.005*1/ν+0.341
R2=0.99
μ/ν
1/ν
0.0
0.1
0.2
0.3
15
30
45


**Fig.S4**

# Supporting Information S1 File captions

**Fig.S1 Specific HAS-FGF21 synthesis rates versus specific growth rates, growth/methanol consumption pattern in HSA-FGF21 production by Mut$^+$ *P. pastoris*.**

●: induction at low cells concentration; ○: at high cells concentration.

**Fig.S2 Energy metabolism patterns $\eta$ in HSA-FGF21 production by a Mut$^+$ *P. pastoris* strain under different induction strategies.**

(A) Categories of NADH distribution $\eta$ ($r^{C}_{NADH}/r^{F}_{NADH}$) under different induction strategies. ░ : 0.8<η<1.0; □: 0.0≤η≤0.8. (B) $\eta$ versus induction time with different induction strategies. ●: initiating induction at ~60 g-DCW/L, run #A; □: at ~100 g-DCW/L, run #B.

**Fig.S3 Monellin fermentation results with fed-batch mode (run #4) and chemostat mode (run #5) at 30°C.**

(A)-(D): run #4 (control, fed-batch mode); (E)-(H): run #5 (chemostat mode).

**Fig.S4 Determination of cells maintenance energy $m_{XE}$ in different fermentation runs.**

**Table S1 Fermentation performance comparisons of HSA-FGF21 production by *P. pastoris* with different induction strategies.**

**Table S2 Major secondary fermentation parameters in HSA-FGF21 production by *P. pastoris* with different induction strategies.**

**Table S3 Selection of dilution rate *D* for the chemostat monellin production using the parameters obtained in run #3 as the reference base.**

**Table S4 Comparison of monellin productivity and concentration in different runs.**
